# CCR9 overexpression promotes T-ALL progression by enhancing cholesterol biosynthesis

**DOI:** 10.3389/fphar.2023.1257289

**Published:** 2023-09-06

**Authors:** Muhammad Jamal, Yufei Lei, Hengjing He, Xingruo Zeng, Hina Iqbal Bangash, Di Xiao, Liang Shao, Fuling Zhou, Quiping Zhang

**Affiliations:** ^1^ Department of Immunology, School of Basic Medical Sciences, Wuhan University, Wuhan, China; ^2^ State Key Laboratory of Agricultural Microbiology, Hubei Hongshan Laboratory, College of Life Science and Technology, Huazhong Agricultural University, Wuhan, Hubei, China; ^3^ Department of Hematology, Zhongnan Hospital of Wuhan University, Wuhan, China; ^4^ Hubei Provincial Key Laboratory of Developmentally Originated Disease, Wuhan University, Wuhan, China

**Keywords:** T-ALL, tissue infiltration, RNA-sequencing, CCR9, SREBF2, cholesterol biosynthesis, simvastatin

## Abstract

**Introduction:** T-cell acute lymphoblastic leukemia (T-ALL) is an aggressive hematological malignancy of the lymphoid progenitor cells, contributing to ∼ 20% of the total ALL cases, with a higher prevalence in adults than children. Despite the important role of human T-ALL cell lines in understanding the pathobiology of the disease, a detailed comparison of the tumorigenic potentials of two commonly used T-ALL cell lines, MOLT4 and JURKAT cells, is still lacking.

**Methodology:** In the present study, NOD-*Prkdc* ^scid^ *IL2rgd* ^ull^ (NTG) mice were intravenously injected with MOLT4, JURKAT cells, and PBS as a control. The leukemiac cell homing/infiltration into the bone marrow, blood, liver and spleen was investigated for bioluminescence imaging, flow cytometry, and immunohistochemistry staining. Gene expression profiling of the two cell lines was performed via RNA-seq to identify the differentially expressed genes (DEGs). CCR9 identified as a DEG, was further screened for its role in invasion and metastasis in both cell lines *in vitro*. Moreover, a JURKAT cell line with overexpressed CCR9 (Jurkat-OeCCR9) was investigated for T-ALL formation in the NTG mice as compared to the GFP control. Jurkat-OeCCR9 cells were then subjected to transcriptome analysis to identify the genes and pathways associated with the upregulation of CCR9 leading to enhanced tumirogenesis. The DEGs of the CCR9-associated upregulation were validated both at mRNA and protein levels. Simvastatin was used to assess the effect of cholesterol biosynthesis inhibition on the aggressiveness of T-ALL cells.

**Results:** Comparison of the leukemogenic potentials of the two T-ALL cell lines showed the relatively higher leukemogenic potential of MOLT4 cells, characterized by their enhanced tissue infiltration in NOD-*PrkdcscidIL2rgdull* (NTG) mice. Transcriptmoe analysis of the two cell lines revealed numerous DEGs, including CCR9, enriched in vital signaling pathways associated with growth and proliferation. Notably, the upregulation of CCR9 also promoted the tissue infiltration of JURKAT cells *in vitro* and in NTG mice. Transcriptome analysis revealed that CCR9 overexpression facilitated cholesterol production by upregulating the expression of the transcriptional factor SREBF2, and the downstream genes: MSMO1, MVD, HMGCS1, and HMGCR, which was then corroborated at the protein levels. Notably, simvastatin treatment reduced the migration of the CCR9-overexpressing JURKAT cells, suggesting the importance of cholesterol in T-ALL progression.

**Conclusions:** This study highlights the distinct tumorigenic potentials of two T-ALL cell lines and reveals CCR9-regulated enhanced cholesterol biosynthesis in T-ALL.

## 1 Introduction

Acute lymphoblastic leukemia (ALL) is an aggressive hematological cancer characterized by the malignant transformation and proliferation of lymphoblasts in bone marrow, blood, and extramedullary tissues (Litzow and Ferrando, 2015; Terwilliger and Abdul-Hay, 2017). Based on the morphology and cytogenetic profiling of the lymphoblast, ALL is classified into B-cell ALL (B-ALL), T-cell ALL (T-ALL). Almost 75% of ALL cases correspond to B-ALL, compared to 10%–15% of T-ALL clinical representation (Wenzinger et al., 2018). Compared to B-ALL, T-ALL is associated with several unfavorable characteristics and a worse prognosis, which generally requires aggressive therapy. Moreover, relapse in clinical T-ALL patients can only be treated with a high dose of chemotherapy along with radiotherapy and bone transplantation (Barrett and Battiwalla, 2010). Although the survival rate of T-ALL patients has significantly improved over the last decades due to advancements in the development of chemotherapies (Pui et al., 2008). However, drug resistance and disease relapse and recurrence offer hindrances to the proper management of T-ALL patients (Follini et al., 2019). This dismal outcome has been associated with the complex nature of the disease, involving the interaction of both genetic and environmental factors to support disease progression and antileukemic drug resistance (Passaro et al., 2016). Therefore, understanding the molecular mechanism underlying T-ALL progression is necessary for the classification of the disease and the development of effective personalized therapeutic strategies against T-ALL.

Cancer cell lines are widely utilized as *in vitro* model systems in biomedical research to investigate disease mechanisms and as a platform for drug discovery (Barretina et al., 2012). Identification of factors associated with T-ALL subtypes and differential clinical outcomes has updated our understanding of the molecular mechanism of T-ALL pathogenesis and target-specific therapeutics (Cordó et al., 2021). In a recent study, single-cell RNA sequencing of thousands of cells isolated from the bone marrow of pediatric T-ALL patients as compared to the healthy pediatric bone marrow revealed deregulated expression of genes associated with cellular growth, proliferation, and metabolic pathways, implicating these genes as oncogenic mediators in T-ALL blasts (Bhasin et al., 2020). Moreover, the molecular characterization of T-ALL genomes based on gene expression pattern, mutation, or copy number variation disclosed additional genomic mutations in the T-cell progenitor impacting the JAK/STAT signaling pathway, protein translation, and epigenetic regulation, thus expanding the current therapeutic option against T-ALL (Girardi et al., 2017). MOLT4 and JURKAT cell lines derived from human T-ALL patients (Minowada et al., 1972; Schneider et al., 1977) are widely utilized models to study the molecular mechanisms underlying T-ALL and develop targeted therapeutic strategies (Dos Santos et al., 2009; Chiarini et al., 2010; Youns et al., 2010; Mezencev and Mcdonald, 2011). Particularly, the MOLT4 cell line, due to its earliest establishment and harboring gene expression patterns, genetic abnormalities, and cellular phenotypes of T-ALL, offers potential to study T-ALL biology and drug development. Likewise, the JURKAT cell line is also important in advancing our understanding of T-ALL pathogenesis as these cells carry several genetic anomalies, including the rearrangement of the T-cell receptor (TCR) and activation of the NOTCH1 signaling pathway, which are common characteristics of T-ALL pathogenesis (Abraham and Weiss, 2004; Gioia et al., 2018). However, cancer cells differ from the primary tumor in a biologically significant manner, and not all the tumor cell lines may recapitulate their annotated cancer type. Earlier studies of multiple cancers have documented discrete molecular profiles of cell lines extracted from the same tumor type, suggesting their dissimilar ability to represent the primary tumors (Jiang et al., 2016; Yu et al., 2019). Notably, analysis of the phosphorylation status of ten signaling pathway proteins with phosopho-specific flow cytometry revealed a higher variability in these proteins across three different T-ALL cell lines under both basal and modulated conditions (Perbellini et al., 2022). Subsequently, T-ALL cell lines, despite their widespread use in cancer biology, differ in their tumorigenic potentials. Moreover, differences between the tumorigenic potentials of MOLT4 and JURKAT cells have not been documented before.

Chemokines and their receptors exhibit great potential in tumor-targeted therapy owing to their active roles in remodeling the tumor microenvironment. The involvement of the CCL25/CCR9 axis in T-ALL is comprehensively summarized by Hong et al. (Hong et al., 2021). Lipid homeostasis is crucial for membrane production and lipid-based protein posttranslational modifications in rapidly proliferating tumor cells to maintain the rapid proliferation rate. Accordingly, leukemia cells reprogram their metabolism by enhancing the *de novo* biosynthesis of cholesterol using different strategies (Ding et al., 2019; Zhao et al., 2019). Studies have shown that chemokines may modulate cholesterol biosynthesis in cancers. A recent study documented the chemokines-induced enhanced cholesterol biosynthesis in the pulmonary tropism of breast cancer cells (Han et al., 2022). However, the role of chemokines in metabolic reshaping in T-ALL remains largely unknown. In this study, we characterized the tumorigenic potentials of both cell lines *in vivo* using NOD PrkdcscidIL2rgdull (NTG) mice. Further, gene expression profiling was carried out to decipher the distinguished transcriptome and signaling pathways and identify several DEGs, including CCR9. Functional genetic studies were conducted to test the role of CCR9 in JURKAT and MOLT4 cells *in vitro*, as well as *in vivo*. In addition, RNA-sequencing of the JURKAT cells overexpressing CCR9 was performed to determine the aberrantly expressed genes and their associated pathways that could contribute to the increased T-ALL tissue infiltration. Inhibition of the cholesterol biosynthesis pathway with simvastatin was carried out to test the antileukemic effect of statin.

## 2 Materials and methods

### 2.1 Cell culture

HEK293T cells and T-ALL cell lines; MOLT-4, and JURKAT cells, were purchased from the American Type Culture Collection, (ATCC, Manassas, VA). HEK293T cells were cultured in DMEM supplemented with 10% fetal bovine serum (FBS, Biological Industries, Israel) and 1% penicillin/streptomycin (Beyotime Biotechnology, China). MOLT-4 and JURKAT cells were cultured in RPMI-1460 media (Biological Industries, Israel) supplemented with 10% fetal bovine serum (FBS, Biological Industries, Israel), 1% L-glutamine (Hyclone, United States), and 1% penicillin/streptomycin (Beyotime Biotechnology, China). The cells in the culture were maintained at 37°C in a humidified atmosphere with 5% CO2.

### 2.2 Lentivirus transduction for stable cell line generation

The plasmid constructs for the overexpression of CCR9, knockdown of CCR9, and a control plasmid were acquired from the Public Protein/Plasmid Library Company. Stable cell line construction was performed following the procedure described in our published study (Zeng et al., 2023). The stable transgenic cell lines obtained after the screening were utilized for subsequent experiments. The efficiency of silencing was verified by RT-qPCR, flow cytometry, and Western blot.

### 2.3 Total cholesterol detection assay

The cells were starved in RPMI media with only 1% FBS for 12 h, followed by seeding of 1×10^6^ cells per well in a 12-well plate. To assess the effect of the CCR9 ligand on the total cholesterol (TC) level, the cells were treated with 100 ng.mL^-1^ of CCL25. After incubation of the cells for 0 h, 24 h, and 48 h, culture medium was collected at the respective time points. The TC in the culture medium was determined according to the total cholesterol assay kit (Abbkine, catalog number KTB2220, China). First, TC working solution was preheated at 37 °C for ∼ 30 min, from which 150 μL was added to each well in a 96-well plate, followed by the addition of 50 μL of measuring standard solution, or RIPA blank solution. After mixing, the reaction was incubated at room temperature for 15 min, and the absorbance was measured at 500 nm wavelength using a microplate reader (TECAN, Switzerland). Finally, a standard curve was generated, and the corresponding total cholesterol concentration of each well was calculated according to the standard curve.

### 2.4 RNA isolation and real-time quantitative PCR (RT-qPCR)

The total cellular RNA was isolated using the TRIzol reagent (Thermo Fisher Scientific, United States) following the manufacturer’s instructions. The mRNA was reverse transcribed into cDNA using the RT-kit following the manufacturer’s guidelines (R323-01; Vazyme, China). The cDNA was amplified with ChamQ SYBR qPCR Master Mix (Q311-02; Vazyme, China) using the Quant Studio 6 Flex Real-Time PCR System (Life Technologies, United States). The expression values of the target gene were normalized according to the reference genes GAPDH and ACTB. The gene expression quantification was evaluated using the 2^−ΔΔCT^ method. The sequence information of the primers used in this study is listed in Table 1.

**TABLE 1:** Primers sequences of genes.

Gene	Forward sequences	Reverse sequences
CCR9	TCG​TGG​TCA​TGG​CTT​GCT​GCT​A	AAG​ACG​GTC​AGG​ACA​GTG​ATG​G
HMGCS1	AAG​TCA​CAC​AAG​ATG​CTA​CAC​CG	TCA​GCG​AAG​ACA​TCT​GGT​GCC​A
MVD	AAG​CGC​GAT​GAA​GAG​CTG​GTT​C	TCC​TCG​GTG​AAG​TCC​TTG​CTG​A
MSMO1	GCT​GCC​TTT​GAT​TTG​TGG​AAC​CT	CTG​CAC​AAC​CAA​AGC​ATC​TTG​CC
HMGCR	GAC​GTG​AAC​CTA​TGC​TGG​TCA​G	GGT​ATC​TGT​TTC​AGC​CAC​TAA​GG
SREBF2	CTC​CAT​TGA​CTC​TGA​GCC​AGG​A	GAA​TCC​GTG​AGC​GGT​CTA​CCA​T
ACTB	CAC​CAT​TGG​CAA​TGA​GCG​GTT​C	AGG​TCT​TTG​CGG​ATG​TCC​ACG​T
GAPDH	CTG​GGC​TAC​ACT​GAG​CAC​C	AAG​TGG​TCG​TTG​AGG​GCA​ATG

shRNA-plasmid	Target sequence information
CCR9-shRNA-1	CCA​GAA​ATC​TTA​TAC​AGC​CAA
CCR9-shRNA-2	GTG​CCG​TTT​CCA​CCA​ACA​TTG
Negative control	GTT​CTC​CGA​ACG​TGT​CAC​GTT

### 2.5 Protein isolation and Western blotting

Isolated cells were lysed with RIPA lysis buffer supplemented with the protease inhibitor PMSF (Beyotime Biotechnology, China) after washing the cells with PBS. The protein content in the cellular lysate was quantified using the BCA kit (Beyotime Biotechnology, China). The lysate samples were separated on SDS-PAGE after boiling at 100°C for 5 min. The proteins on the gel were cold transferred to a polyvinylidene difluoride membrane (Millipore, United States) for 2 h. After transfer, membrane blocking was done with 5% milk dissolved in PBST, followed by incubation with the target primary antibody for overnight at 4°C with gentle shaking. The membrane was washed and probed with secondary antibodies (Proteintech, China) for 1 h. The protein signal in the membrane was detected using an ultra-high-sensitivity ECL kit (MedChemExpress, United States). Antibodies: CCR9 (Abcam, ab32556), HMGCS1 (Proteintech, 17643-1-AP), MSMO1 (Immunoway, YT7443), HMGCR (HUABIO ET1702-41), MVD (Proteintech, 15331-1-AP), ACTIN (HUABIO, ET1702-52), GAPDH (ABclonal, AC002), HRP-conjugated anti-mouse IgG secondary antibodies (Proteintech, SA00001-1), HRP-conjugated anti-rabbit IgG secondary antibodies (Proteintech, SA00001-2).

### 2.6 Transwell migration/chemotaxis, and invasion assay

Transwell migration/chemotaxis and invasion assay were carried out in 24-well transwell chambers (Corning) using 5 μm pore size polycarbonate insets (Corning, United States). For transwell migration assay, culture with 1×10^5^ suspended in cells in 1% FBS medium was seeded in the upper chamber. 600 μL culture medium with 10% FBS was added into the lower chamber. For chemotaxis assay, 100 ng/mL CCL25 in RPMI 1640 with 1% FBS was placed in the lower well, and cell suspension containing 1 × 10^5^ in 1% FBS was added to the upper well of the chamber. For the invasion assay, the transwell chamber was coated with 100 μL of 1:7 diluted Matrigel (BD Biosciences, United States). Cells (1–2 × 10^5^) suspended in 2% FBS medium were seeded onto the upper chambers followed by addition of either 10% FBS medium or 2% FBS medium with CCL25 to the bottom chambers. The cells were allowed to migration for 24 h at 37°C. The metastatic cells were counted using an inverted microscope with a hemacytometer. The results of migration/invasion assays are expressed as fold values.

For the chemotaxis assay after simvastatin treatment, cells were treated with different concentrations of simvastatin 3 μM, 6 μM, 12 μM and 24 μM, (Sigma, S6196) for 24 h. Subsequently, 1×10^5^ cells in 1% FBS medium were seeded in the upper chamber, and 600 μL medium with 10% FBS was added to the lower chambers. After 12 h, the cells in the lower well were recovered and counted using a Neubauer chamber.

### 2.7 Flow cytometry

Cells were washed and resuspended at a concentration of 1×10^6^ cells per 100 μL in PBS. Cells were stained with the corresponding monoclonal antibodies as per the manufacturer’s instructions, then washed and analyzed with FACS Aria III flow cytometer (BD, United States). The FACS data were analyzed with FlowJo (BD Bio, United States). CCR9 (BD Pharmingen, United States), Hu CD45 APC HI30 100Tst (BD Pharmingen, United States), and DAPI (BD Pharmingen, United States).

### 2.8 RNA sequencing (RNA-Seq) and bioinformatics analysis

MOLT4 cells, JURKAT cells, JURKAT cells overexpressing CCR9 (Oe-CCR9-JURKAT) cells, and the GFP control were used for the RNA-sequencing. We performed all the RNA-seq in biological replicates. The total RNA of the corresponding cells was extracted following the aforementioned protocol. The integrity of RNA was assessed by 1% agarose gel electrophoresis, and integrity was validated by running the RNA Nano 6000 Assay Kit of the Bioanalyzer 2100 system (Agilent Technologies, United States). A total of 1 μg of RNA per sample was used as input material to generate the RNA-sequencing library using the Illumina NEBNext^®^ UltraTM RNA Library Prep Kit following the manufacturer’s protocol. The library was sequenced on an Illumina Novaseq platform and 150 bp paired-end reads were generated. After pretreatment of the raw reads (filtering and QC), clean reads were aligned to the human reference genome using Hisat2 v2.0.5. After mapping to the reference genome, the read numbers mapped to each gene were initially counted with FeatureCounts (v 1.5.0-p3), followed by the calculation of FPKM values for each gene based on the length of the gene and the read counts mapping to it. The read counts were adjusted by the edgeR program package through one scaling normalized factor. Next, the edgeR R package (version 3.22.5) was used to perform differential expression analysis between the conditions. Adjacent *p*-values were calculated following Benjamini and Hochberg’s approach. The corrected *p*-values of <0.05 and log2FC > 1 were set as the threshold for defining a significant differential gene expression.

The enrichment of the DEGs in the Gene Ontology (GO), KEGG pathway, Reactome pathway, DisGeNET pathway, and disease ontology (DO) pathway was implemented by the clusterProfiler R package (3.8.1) in R-studio. Pathways associated with the DEGs with *p*-values ≤ 0.05 were regarded as significant. STRING database was used to construct the network of genes and was visualized with Cytoscape (v.3.9.1).

GSE48558 and GSE26713 datasets used in this study were retrieved from the Gene Expression Omnibus (http://www.ncbi.nlm.nih.gov/geo). GSE48558 dataset contains gene expression data obtained from normal and malignant hematopoietic cells. GSE26713 dataset contains the gene expression data of 117 human T-ALL bone marrow samples and 7 normal individual bone marrow samples. Additionally, GEO2R (https://www.ncbi.nlm.nih.gov/geo/geo2r/) was used to compare the expression of the genes between leukemia and normal conditions. UALCAN database (http://ualcan.path.uab.edu/index.html), containing the cancer genome atlas (TCGA) gene expression data, was utilized to determine the expression of genes in various cancers.

### 2.9 Xenotransplantation experiments

NTG mice (female, 4–6 weeks old, Institute of Model Animals, Wuhan University, China) were reared in a sterile animal facility on a 12-h light and dark cycle. The mice were treated in accordance with European Union guidelines and with the approval of the Medical Ethics Committee of Wuhan University School of Medicine (Permit Number: WP20220022). MOLT-4 and JURKAT cells constitutively expressing the luciferase gene under a ubiquitous promoter were tail injected into NTG immunocompromised mice (1×10^7^ cells per mouse). Simultaneously, PBS was used as a control. Next, JURKAT cell line overexpressing CCR9 (OeCCR9-JURKAT) was injected into the NTG. For comparison GFP expressing–JURKAT cells (GFP-JURKAT) was used. The imaging system Xtreme BI (Bruker, United States) was used to image bioluminescence *in vivo*. Mice were intraperitoneally injected with 150 mg/kg D-Luciferin and Potassium Salt (Yeason, China). Engraftment and disease progression were analyzed by the assessment of body weight, peripheral blood smear, flow cytometry, IHC staining, bioluminescence imaging, and mouse organ examination.

### 2.10 Wright’s Giemsa (WG) staining

The slides of bone marrow (BM) and peripheral blood (PB) from mice were stained with Wright’s Giemsa stain solution (Solarbio, G1020) according to the manufacturer’s instructions and were then observed or photographed under the microscope (Nikon, Japan).

### 2.11 Hematoxylin-eosin (HE) staining

The tissue samples from the brain, liver, and spleen of mice were fixed with 4% paraformaldehyde and then embedded in paraffin. The paraffinized tissues were sliced into sections. The preserved tissue sections were deparaffinized, rehydrated, and finally stained with hematoxylin and eosin using the HE Staining Kit (Servicebio, G1003). All the procedures were performed according to the manufacturer’s instructions. All the finished sections were observed and photographed under the microscope (Nikon, Japan).

### 2.12 Immunohistochemical (IHC) staining

The IHC staining assay was performed as described in the previous study (Lei et al., 2022). Briefly, the microarray tissue samples were dewaxed with xylene twice for 15 min and rehydrated with an increased concentration of ethanol for 5 min each. Sodium citrate buffer was used for antigen retrieval, and the samples were heated at 100°C followed by inhibition of the endogenous peroxidase activity with 3% H2O2. Then the sections were blotted with the primary antibodies against CD45 (Servicebio, GB113885) and SREBF2 (Proteintech 14508-1-AP). After washing with PBS, the tissue sections were incubated with a secondary antibody (Servicebio, G1215). The histochemistry score (H-score) was calculated to assess the IHC results. H-score = (percentage of cells of weak intensity × 1) + (percentage of cells of moderate intensity × 2) + (percentage of cells of strong intensity × 3).

### 2.13 Statistical analysis

The statistical analysis and data visualization was performed with GraphPad Prism (v. 8.0). Unpaired *t*-test and one-way ANOVA followed by Dunnett’s test were employed. A *p*-value < 0.05 was considered statistically significant.

## 3 Results

### 3.1 MOLT4 cells exhibit remarkably increased tumorigenic potential *in vivo*

**FIGURE 1:**
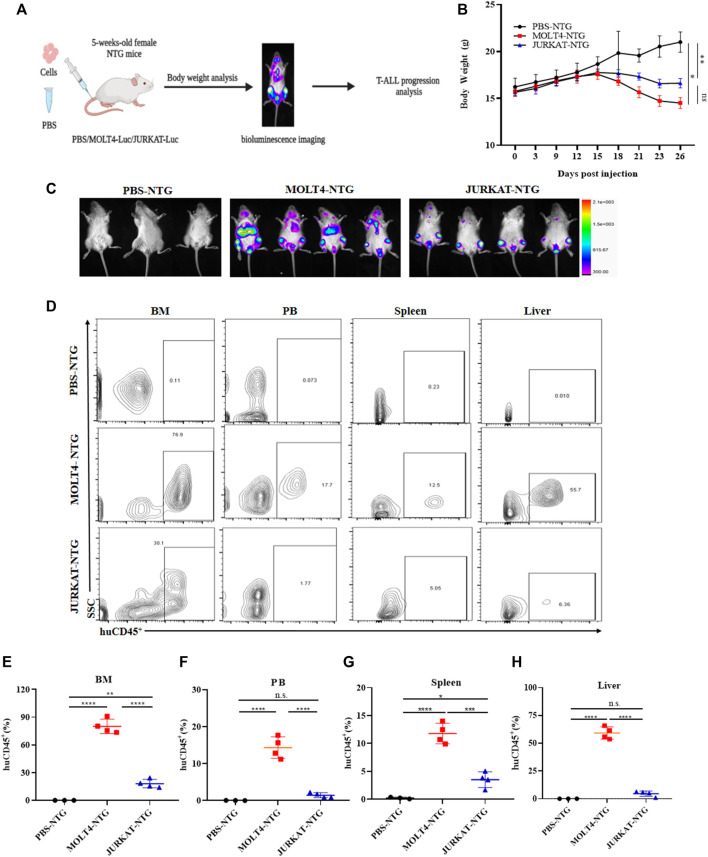
MOLT4 exhibits remarkably increased tumorigenic potential ***in vivo*. (A)** Schematic representation of the strategy used in this study, NTG mice (*n* = 5) in each group were injected with MOLT4 and JURKAT cells expressing the luciferase gene as compared to the PBS control **(B)** curve representing the actual average weight of the mice during the course of 26 days of incubation. **(C)** Bioluminescence imaging was conducted 26 days after the injection. **(D)** Flow cytometry analysis of MOLT4 and JURKAT cells in peripheral blood, bone marrow, spleen, or liver of NTG mice gated on human CD45. **(E–H)** The percentage of cells represents MOLT4 and JURKAT cells over total gated cells, respectively. Each symbol represents data for an individual mouse. Data represent the mean ± SD. ∗*p* < 0.05, ∗∗*p* < 0.01, ∗∗∗*p* < 0.0001 by 1-way ANOVA. A representative experiment from three independent experiments is shown.

**FIGURE 2:**
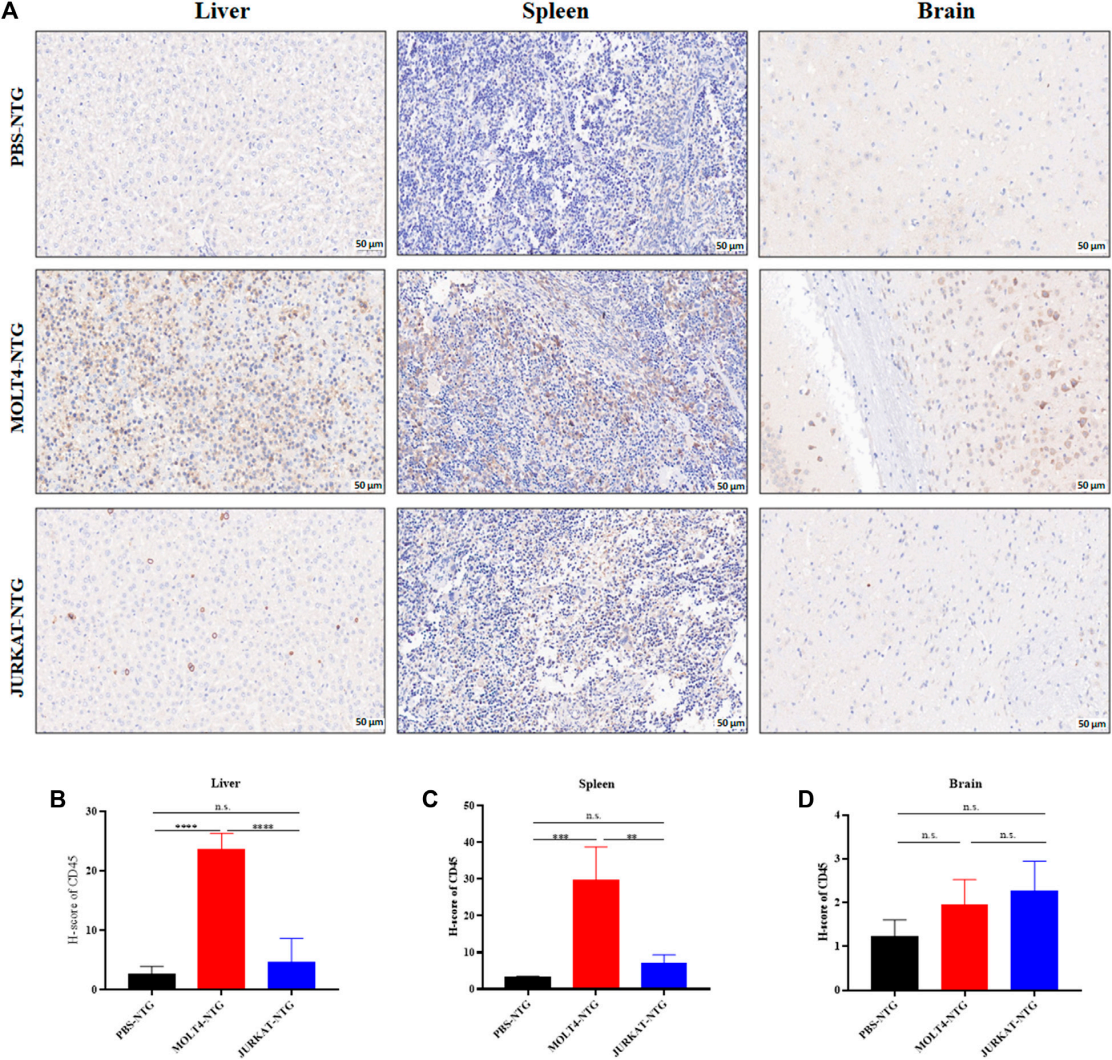
MOLT4 cells exhibit an increased pattern of organ infiltration compared to JURKAT cells. **(A)** Representative images of the IHC analysis of CD45^+^ cells in liver, spleen, and brain tissue sections of mice injected with PBS, MOLT4, and JURKAT, respectively. **(B)** H-scores of the CD45^+^ cells in the liver of PBS-NTG, MOLT-NTG, and JURKAT-NTG mice. **(C)** H-scores of the CD45^+^ cells in the spleen of PBS-NTG, MOLT-NTG, and JURKAT-NTG mice. **(D)** H-scores of the CD45^+^ cells in the brain of PBS-NTG, MOLT-NTG, and JURKAT-NTG mice. Data represent the mean ± SD. ∗*p* < 0.05, ∗∗*p* < 0.01, ∗∗∗*p* < 0.0001 by 1-way ANOVA.

To characterize the tumorigenic potentials of the two T-ALL cell lines, an *in vivo* investigation with the NTG mice model was performed following the workflow outlined in Figure 1A. To this end, JURKAT and MOLT4 cells expressing the luciferase gene and PBS were injected intravenously (i.v.) into the immunodeficient NTG mice. The mice were maintained for 26 days, imaged, and sacrificed for subsequent pathogenic examinations. A significant body weight reduction was observed in mice bearing MOLT4 cells (MOLT4-NTG), represented by the red line, as compared to the group of mice injected with JURKAT cells (JURKAT-NTG) and the PBS control (PBS-NTG) (Figure 1B). Likewise, the bioluminescence imaging presented a significantly increased expansion of the cells in MOLT4-NTG, as shown by the enhanced luciferase signal, compared to JURKAT-NTG and PBS-NTG mice (Figure 1C). Next, we determined the organ weight of the mice in three groups and found a robust increase in liver and spleen weight of MOLT4-NTG mice as compared to JURKAT-NTG and PBS-NTG mice (Supplementary Figures S1A, B), showing a robust increased tumor formation ability of MOLT4 cells. We used flow cytometry to detect the dissemination of JURKAT and MOLT4 cells in multiple organs, respectively, by gating on human CD45. Consistently, a profound increase in the percentage of CD45^+^ cells was observed not only in BM and PB but also in the liver and spleen of MOLT4-NTG mice, showing a remarkably increased organ infiltration of MOLT4 cells as compared to JURKAT cells (Figures 1D–H). Moreover, HE and WG staining of the tumor sections displayed an increased tumor infiltration of the MOLT4 cells to the liver, spleen, brain, bone marrow, and peripheral blood as compared to the JURKAT cells (Supplementary Figure S1C). To corroborate these findings, we performed IHC of the brain, liver, and spleen tissue sections acquired from NTG mice of the two groups and found an elevated number of CD45^+^ cells in the liver of MOLT4-NTG mice as compared to liver obtained from JURKAT-NTG and PBS-NTG mice (Figures 2A, B). We observed a similar trend in the spleen (Figures 2A, C). However, no striking difference in the number of CD45^+^ cells in brain of the different mice groups was observed (Figures 2A, D). Taken together, these results support the notion that MOLT4 cells display a relatively higher tissue infiltration ability as compared to JURKAT cells.

### 3.2 Transcriptome profiling of T-ALL cell lines revealed different DEGs involved in vital cellular pathways

**FIGURE 3:**
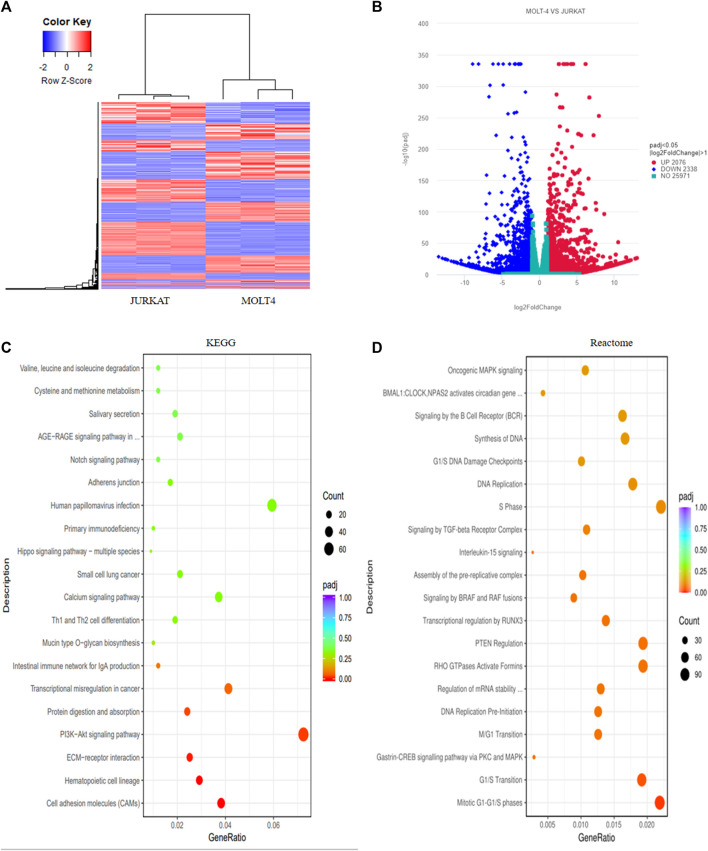
Transcriptome profiling of T-ALL cell lines revealed different differentially expressed genes involved in vital cellular pathways. **(A)** The heatmap represents the expression pattern of the differentially expressed genes (DEGs) between the MOLT4 and JURKAT cells. Red and blue represent upregulated and downregulated genes, respectively. **(B)** The volcano plot represents the distribution of DEGs obtained between MOLT4 and JURKAT cells following the criteria (log2FC > 1, padj < 0.05). Red and blue dots refer to upregulated and downregulated genes, respectively. **(C)** KEGG pathway enrichment analysis of the DEGs: the dot represents the number of DEGs count enriched in a particular pathway, whereas the color represents the *p*-value. **(D)** Enrichment of the DEGs in the GO in the Reactome pathway enrichment database.

We performed RNA-sequencing of MOLT4 and JURKAT cell lines to characterize the differences in the gene expression profiles and the associated pathways that cause the differences in the tumorigenic potential of the two cell types. We identified 4414 DEGs, including 2076 upregulated genes using the criteria DESeq2, padj = < 0.05, and Log2 FC > 1 (Supplementary Table S1) and 2338 downregulated genes using the criteria DESeq2, padj = < 0.05, and log2 FC > −1 in the R program (Supplementary Table S2). The differential expression of genes between the two cell lines is represented as a heatmap (Figure 3A), and the distribution of the DEGs between the cells is illustrated in the volcano plot (Figure 3B). KEGG pathway enrichment analysis displayed the involvement of the DEGs in the PI3K-Akt signaling pathway, cell adhesion molecules, and hematopoietic cell lineage (Supplementary Table S3, Figure 3C). The Reactome database integrates the biological and molecular pathways of model organisms, including humans (Croft et al., 2010). The Reactome pathway enrichment analysis with a padj > 0.05 as a threshold displayed the association of the DEGs with various vital cell cycle-related pathways, including mitotic G1-G1/S phases, G1/S transition, M/G1 transition, and tumor suppressor pathways, including PTEN regulation and signaling by the TGF-beta receptor complex (Figure 3D). The DisGeNET database is a collection of resources providing information about the gene and its variants in human diseases (Piñero et al., 2020). The DisGeNET gene ontology (GO) analysis showed the abundance of genes in leukemic pathways such as precursor cell lymphoblastic leukemia, leukemia-T cell, and adult T-cell lymphoma/leukemia (Supplementary Figure S2A). The gene ontology function of the DEGs revealed the enrichment of these genes in various important biological pathways such as T-cell activation, actin cytoskeleton, transcription factor activity, DNA binding, and axon development (Supplementary Table S4 and Supplementary Figure S2B). Moreover, the biological function (BF) category of the GO analysis revealed the enrichment of the DEGs in T-cell activation, axon development, and embryonic organ morphogenesis. Regarding the cellular component, DEGs were enriched in the actin cytoskeleton, receptor complex, and extracellular matrix component. Whereas the DEGs were associated with various enzymatic activities, including protein tyrosine kinase, transcriptional activation, enzyme inhibition, and signal transduction, in the molecular function (MF) category of the GO analysis (Supplementary Figure S2C).

These findings highlight the distinct gene expression profiles and high signaling variability across the two cell lines. Moreover, enrichment in various important biological pathways, such as T-cell development and activation, may lead to the distinct developmental fates and subsequent tumorigenic potentials of the two cell lines.

### 3.3 The aberrant expression of CCR9 affected the metastasis and invasion of T-ALL cell lines

**FIGURE 4:**
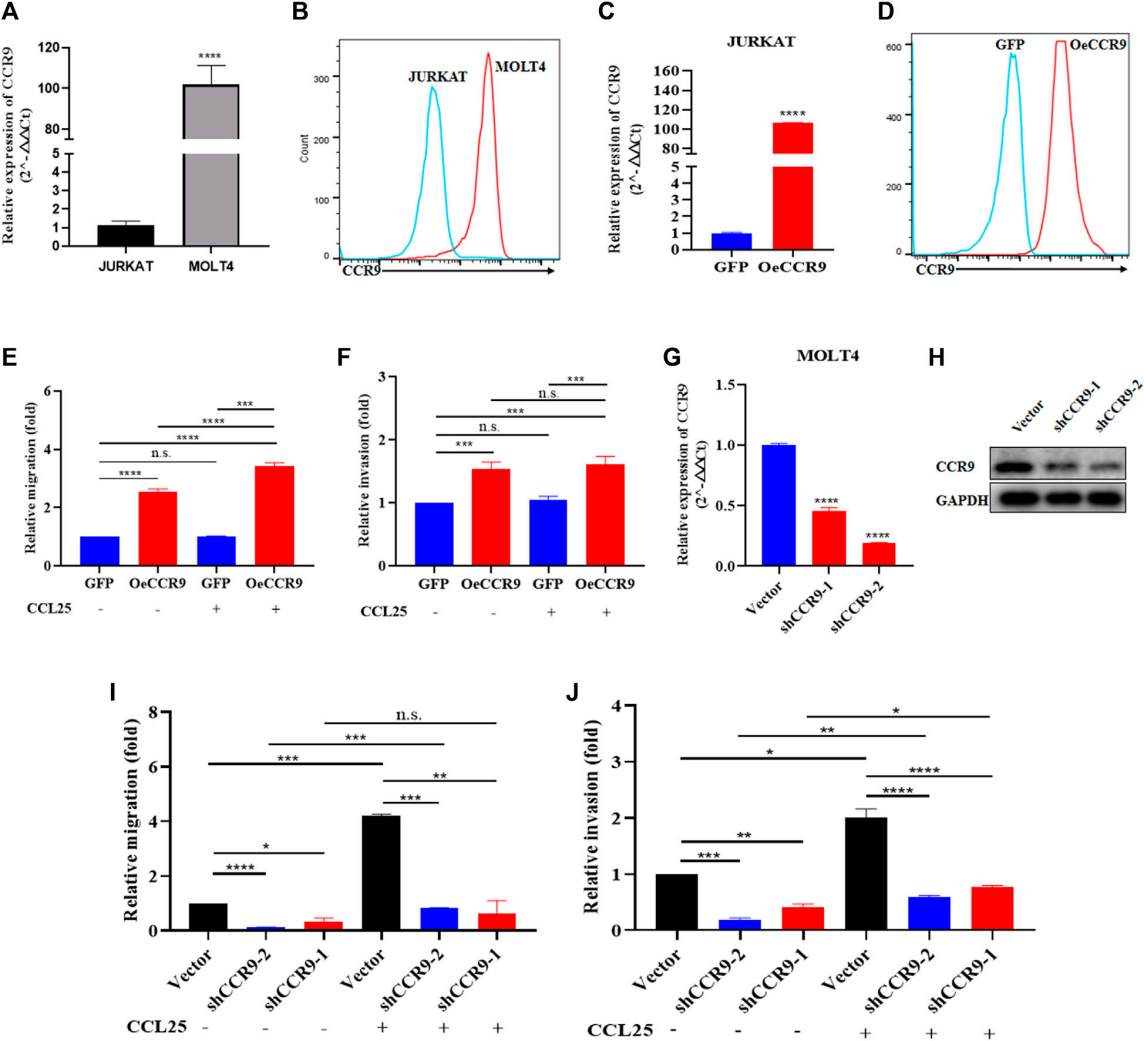
The aberrant expression of CCR9 affected the metastasis and invasion of T-ALL cell lines. **(A)** Quantification of CCR9 expression in MOLT4 and JURKAT cell lines on the mRNA level. **(B)** Comparative expression analysis of CCR9 between MOLT4 and JURKAT cell lines using flow cytometry. **(C)** Confirmation of CCR9 overexpression in JURKAT cells via qRT-PCR. **(D)** Confirmation of CCR9 overexpression in JURKAT cells via flow cytometry. **(E)** The relative migration of JURKAT with overexpressed CCR9 as compared to the vector control with and without stimulation with CCL25. Cells in suspended in 1% FBS were added to the upper chamber. Transwell chambers containing either 10% FBS medium or 2% FBS medium with CCL25 (100 ng/mL) in the bottom chambers were used for cell migration and chemotaxis assays, respectively. Migrated cells in the bottom chamber were quantified after 24 h. **(F)** Transwell chambers coated with Matrigel (1:7 ratio) and FBS medium with and without CCL25 (100 ng/mL) in the bottom chambers were used for cell invasion assays. Invaded cells in the bottom chamber were quantified after 24 h. The results of assays are expressed as fold value. The relative invasion of JURKAT cells overexpressing CCR9 as compared to the vector control with and without stimulation with CCL25. **(G, H)** shRNA-mediated stable silencing of CCR9 in MOLT4 cells, two different shRNAs with different silencing effects are shown. **(I)** ShRNA-2-mediated CCR9 silencing yielded a reduction in the migration of MOLT4 cells. **(J)** A similar repressing effect of the shRNA2-mediated silencing of CCR9 on the invasion of MOLT4 is evident. Data represent the mean ± SD. ∗*p* < 0.05, ∗∗*p* < 0.01, ∗∗∗*p* < 0.0001 by 2-tailed Student’s *t*-test or 1-way ANOVA. A representative experiment from three independent experiments is shown.

In order to get crucial candidate genes in T-ALL, we set a stringent criterion (log2FC > 5 and padj value = <0.05) to get significantly upregulated genes. We found C-C chemokine receptor 9 (CCR9) with a log2FC = 7.941006 and padj = 2.58E-253 as one of the crucial genes with clinical relevance in T-ALL (Supplementary Table S5). To explore the role of CCR9 in T-ALL, we analyzed published T-ALL GSE datasets (GSE33315, GSE48558, and GSE13159) and observed an elevated expression of CCR9 in clinical samples and T-ALL cell lines (Supplementary Figure S3). Next, we determined the expression of CCR9 in both cells and noted that MOLT4 cells displayed an increased expression of CCR9 (Figures 4A, B). We then investigated the role of CCR9 in T-ALL by upregulating CCR9 in JURKAT cells (OeCCR9-JURKAT) (Figures 4C, D). Next, to assess whether the upregulation of CCR9 influences the migration and invasion, and chemotaxis abilities of the T-ALL cells, *in vitro* transwell migration/chemotaxis and invasion assays were performed. A pictorial representation of the mechanism of these assays is given in Supplementary Figure S4. We found that migration of the JURKAT cells through the chamber increased when CCR9 was upregulated as compared to the GFP control (Figure 4E). C-C chemokine ligand 25 (CCL25) a ligand for CCR9, orchestrates the trafficking of lymphocytes and induces migration of CCR9^high^ T-ALL cells, polarization, and microvilli absorption, enhancing T-ALL cell infiltration (Tu et al., 2016). To check whether CCR9 ligand impact cell migration and invasion, CCL25 was added to the cell culture media in the bottom chambers to stimulate the cells. We observed a synergistic effect of CCL25 addition on the migration of CCR9-overexpressing JURKAT cells (Figure 4E). Next, the CCR9 overexpressing JURKAT cells were checked for their invasive properties by assessing their migration through a Matrigel-coated transwell chamber. Notably, the invasive capacity of the JURKAT cell was increased with the overexpression of CCR9 as compared to GFP. (Figure 4F), whereas no remarkable difference in the invasion of OeCCR9-JURKAT cells was observed with the addition of CCL25 (Figure 4F). Since MOLT4 cells constitutively express CCR9, we aimed to ascertain its role in MOLT4 cells by repressing its expression. To this end, we stably silenced CCR9 by using two different short hairpin RNAs (shRNAs) (Table 1) (Figures 4G, H) and analyzed their influence on the migration and invasion of MOLT4 cells. Of note, reduced migration and invasion of MOLT4 cells upon the silencing of CCR9 with shRNA-2 were observed (Figures 4I, J). We observed that the addition of CCL25 facilitated the migration and invasion of the MOLT4 cells in the control (vector) group, suggesting the dependence of CCL25 on its cognate receptor, CCR9. These findings highlight the functional role of CCR9 in the aggressiveness of the leukemic cells and support the notion that the aberrant expression of CCR9 in turn could potentially contribute to distinct T-ALL outcomes.

### 3.4 The overexpression of CCR9 increased the tumorigenic potential of JURKAT cells *in vivo*

**FIGURE 5:**
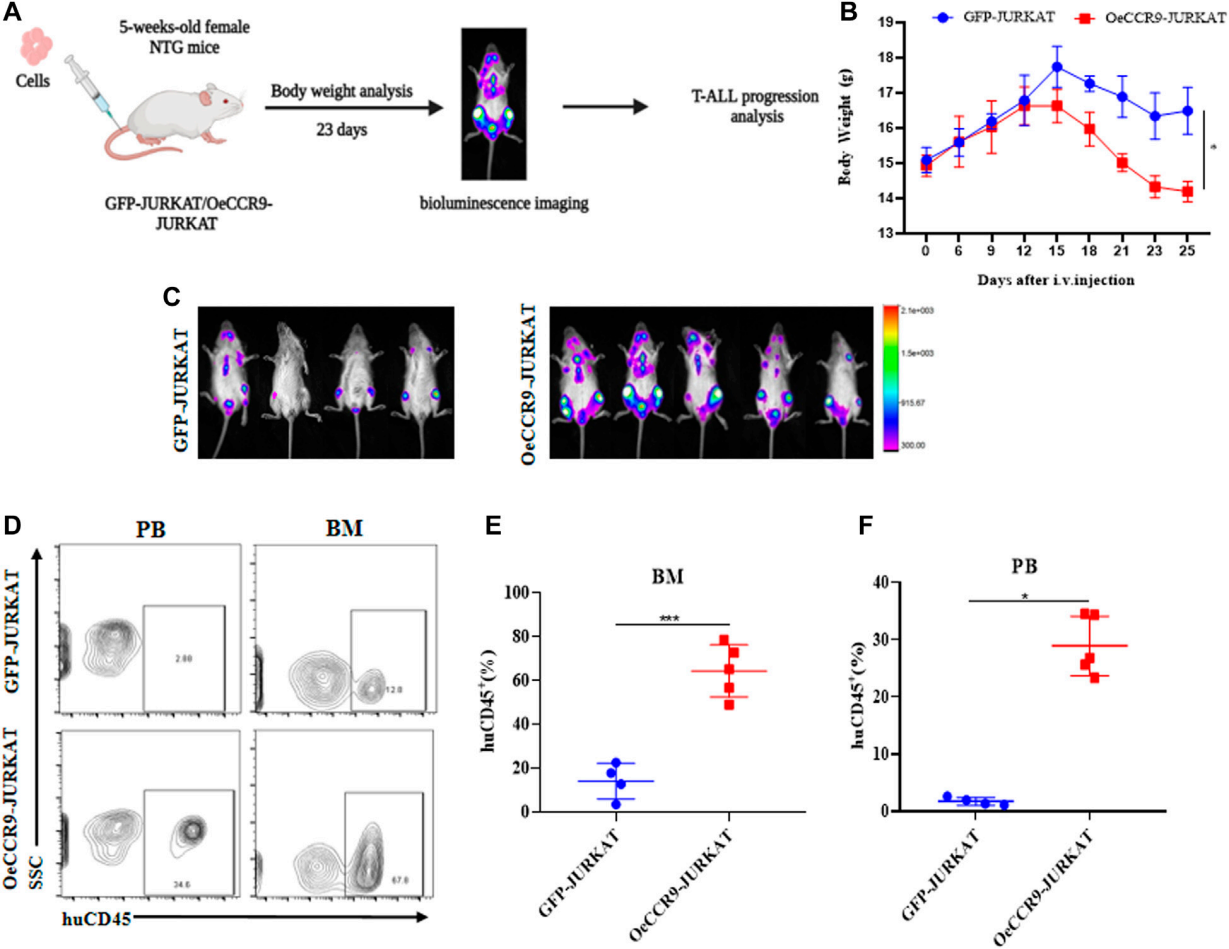
Overexpression of CCR9 increased the tumorigenic potential of JURKAT cells *in vivo*. **(A)** Schematic representation of the strategy used in this study, mice were divided into two groups, and NTG mice (*n* = 5) in each group were injected with OeCCR9-JURKAT cells and GFP-JURKAT cells as controls. The mice were maintained for 23 days, imaged for luciferase signaling, sacrificed, and subsequently analyzed for T-ALL progression. **(B)** Body weight analysis of the NTG mice in the two groups. The data is represented as the actual average weight measured at different days **(C)** Bioluminescence imaging of the OeCCR9-JURKAT-NTG and GFP-JURKAT-NTG mice was performed 23 days post-injection. **(D–F)** Flow cytometry analysis of CD45^+^ cells in peripheral blood and bone marrow.

**FIGURE 6:**
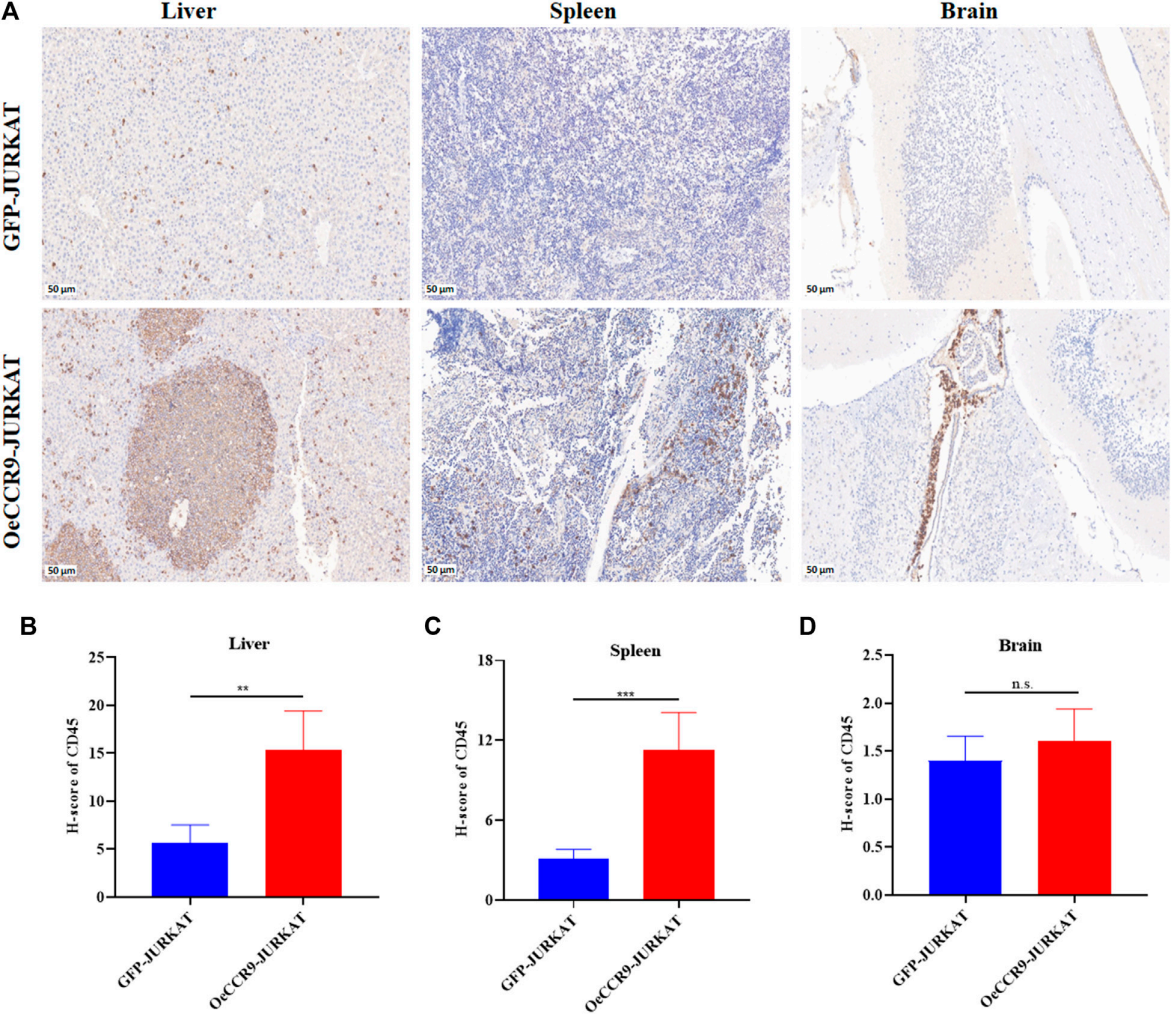
OeCCR9-JURKAT cells exhibit an increased pattern of organ infiltration. **(A)** Representative images of the IHC analysis of CD45^+^ cells in liver, spleen, and brain tissue sections of OeCCR9-JURKAT-NTG and GFP-JURKAT-NTG mice, respectively. **(B)** H-scores of the CD45^+^ cells in the liver of OeCCR9-JURKAT-NTG and GFP-JURKAT-NTG mice. **(C)** H-scores of the CD45^+^ cells in the spleen of OeCCR9-JURKAT-NTG and GFP-JURKAT-NTG mice. **(D)** H-scores of the CD45^+^ cells in the brain of OeCCR9-JURKAT-NTG and GFP-JURKAT-NTG mice. Data represent the mean ± SD. ∗*p* < 0.05, ∗∗*p* < 0.01, ∗∗∗*p* < 0.0001 by 1-way ANOVA.

To corroborate the *in vitro* findings of the oncogenic function of CCR9, we tested its role *in vivo* by xenotransplanting the OeCCR9-JURKAT into NTG mice. To this end, 5-week-old NTG mice were subjected to i.v. injection of OeCCR9-JURKAT cells (OeCCR9-JURKAT-NTG) as compared to JURKAT cells expressing GFP (GFP-JURKAT-NTG). The mice were reared for 25 days before tumor imaging for luciferase signal intensity and disease progression analysis (Figure 5A). Consequently, a robust decline in body weight was observed in OeCCR9-JURKAT-NTG mice as compared to GFP-JURKAT-NTG mice (Figure 5B). We observed an enhanced luciferase signal in mice harboring OeCCR9-JURKAT, showing the abundance of CCR9-overexpressing JURKAT cells compared to the GFP-JURKAT cells (Figure 5C). Moreover, analysis of the organ weight showed a remarkable increase in spleen (Supplementary Figure S5A) and liver weight (Supplementary Figure S5B), marking increased organ infiltration of OeCCR9-JURKAT cells. To assess the increased infiltration of OeCCR9-JURKAT cells that could lead to enhanced disease progression, the absolute number of CD45^+^ cells in BM and PB was quantified with flow cytometry. Consistently, we observed a noticeable increase in the number of CD45^+^ cells in the BM and PB of OeCCR9-JURKAT-NTG mice as compared to the GFP-JURKAT-NTG group (Figures 5D–F). HE and WG staining also portrayed elevated T-ALL formation in the PB, BM, spleen, and liver (Supplementary Figure S5C). Next, we performed IHC of CD45^+^ cells in the liver, spleen, and brain tissue sections obtained from mice in the two groups. We observed an elevated number of CD45^+^ cells in the liver and spleen of OeCCR9-JURKAT-NTG mice as compared to the liver tissue section obtained from GFP-JURKAT-NTG mice (Figures 6A–C). However, no striking difference in the number of CD45^+^ cells infiltrating the brain was observed (Figures 6A, D). Based on these findings from our *in vitro* and *in vivo* investigations, CCR9 promotes the infiltration of T-ALL cells into the liver and spleen.

### 3.5 The overexpression of CCR9 was associated with increased cholesterol biosynthesis in T-ALL

**FIGURE 7:**
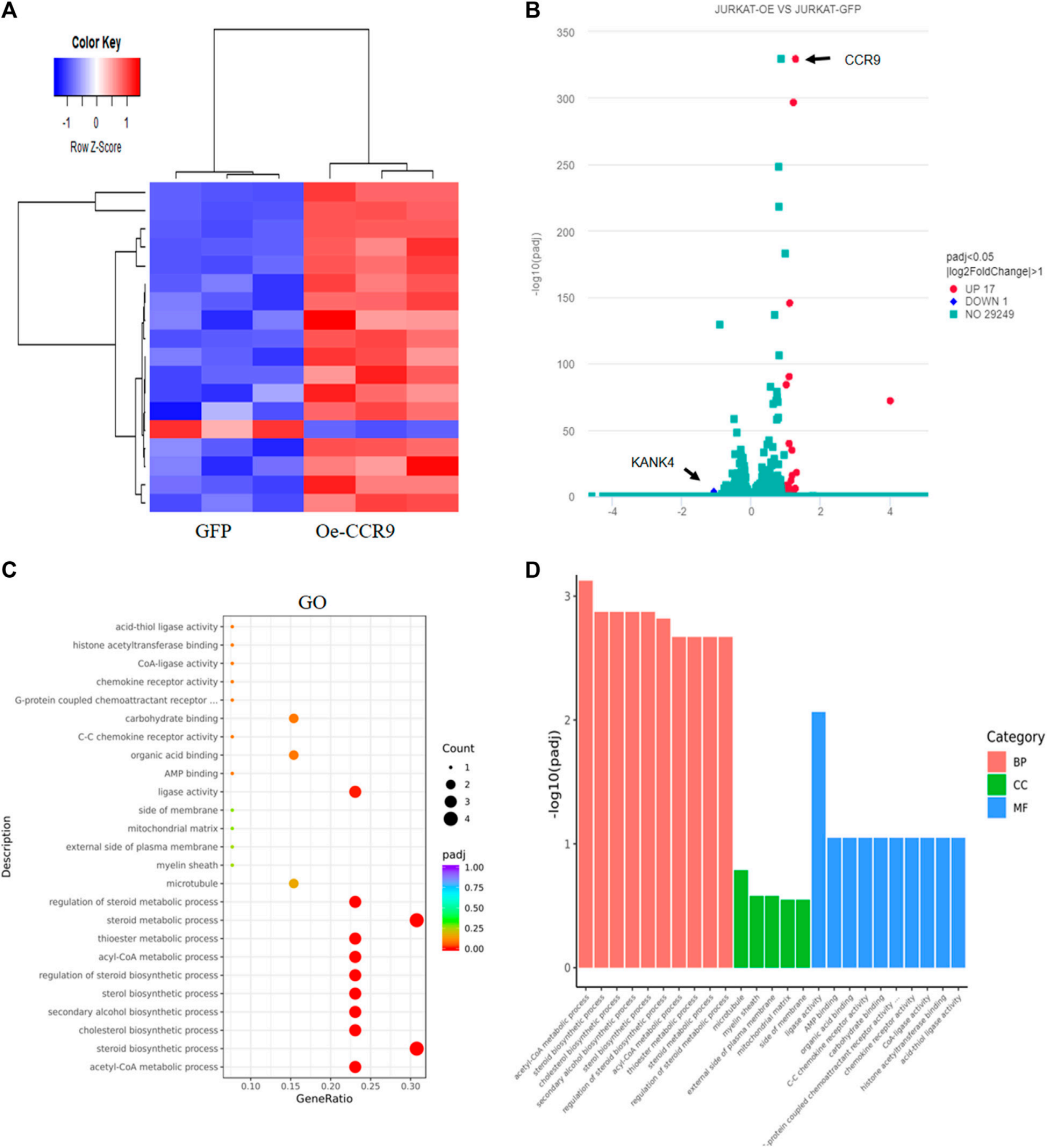
Transcriptome profiling of the Oe-CCR9-JURKAT cells revealed differentially expressed genes associated with metabolic pathways. **(A)** A heatmap representing the expression pattern of the DEGs between OeCCR9-JURKAT and GFP-JURKAT cells. Red and blue represent upregulated and downregulated genes, respectively. **(B)** The volcano plot represents the distribution of DEGs obtained between OeCCR9-JURKAT and GFP-JURKA cells following the criteria (log2FC > 1, padj<0.05). Red and blue dots refer to upregulated and downregulated genes, respectively. **(C)** GO pathway enrichment analysis of the DEGs, the dot represents the number of DEGs count enriched in a particular pathway, whereas the color represents the *p*-value. **(D)** The enrichment of DEGs in a cellular component, molecular function, and biological process categories in GO function.

To gain insights into the molecular mechanism governing the aggressive T-ALL phenotype conferred as a result of the overexpression of CCR9, we performed RNA-sequencing of the OeCCR9-JURKAT and GFP-JURKAT cells. Using the criteria DESeq2, padj = < 0.05, and log2 FC > 1, we obtained a total of 18 DEGs, including 17 upregulated genes and 1 downregulated gene (Table 2) (Figure 7A). A 4-fold overexpression of CCR9 is shown in the volcano plot, thus confirming the reliability of our RNA sequencing data (Figure 7B). We performed the DisGeNET GO enrichment analysis to get the disease phenotype associated with CCR9 overexpression and found its enrichment in leukemic pathways including precursor cell lymphoblastic leukemia, acute promyelocytic leukemia, and acute lymphocytic leukemia (Supplementary Figure S6A). The KEGG function revealed the association of DEGs with metabolic pathways including carbon metabolism, biosynthesis of amino acids, and biosynthesis of steroids (Supplementary Figure S6B). Moreover, when investigated in GO function, DEGs were found supplemented in the lipid biosynthesis pathways, including the steroid biosynthetic process, cholesterol biosynthetic process, steroid metabolic process, and regulation of the steroid metabolic process (Supplementary Table S6; Figures 7C, D).

**TABLE 2:** DEGs of OeCCR9.

Gene_Id	Log2FC	p-value	p-adj	Gene name
ENSG00000112972	1.272596	0	0	HMGCS1
ENSG00000064886	1.21112	7.77E^-301^	3.03E^-297^	CHI3L2
ENSG00000052802	1.101884	1.75E^-149^	2.91E^-146^	MSMO1
ENSG00000181577	1.084427	6.27E^-94^	6.65E^-91^	C6orf223
ENSG00000167508	1.005813	1.19E^-87^	1.16E^-84^	MVD
ENSG00000173585	4.004182	1.82E^-75^	1.25E^-72^	CCR9
ENSG00000130707	1.082549	3.59E^-43^	1.68E^-40^	ASS1
ENSG00000225855	1.164233	4.23E^-38^	1.70E^-35^	RUSC1-AS1
ENSG00000120738	1.295977	5.11E^-21^	1.17E^-18^	EGR1
ENSG00000134594	1.170273	1.65E^-18^	3.15E^-16^	RAB33A
ENSG00000131069	1.142529	1.06E^-14^	1.50E^-12^	ACSS2
ENSG00000110848	1.016023	4.57E^-11^	4.56E^-09^	CD69
ENSG00000183604	1.076263	2.93E^-09^	2.14E^-07^	SMG1P5
ENSG00000223901	1.267168	2.12E^-08^	1.30E^-06^	AP001469.1
ENSG00000274536	1.131767	3.17E^-07^	1.39E^-05^	AL034397.3
ENSG00000131044	1.007419	1.36E^-06^	4.94E^-05^	TTLL9
ENSG00000132854	−1.07934	8.30E^-06^	0.000244	KANK4
ENSG00000170390	1.136976	2.34E^-05^	0.000606	DCLK2

**FIGURE 8:**
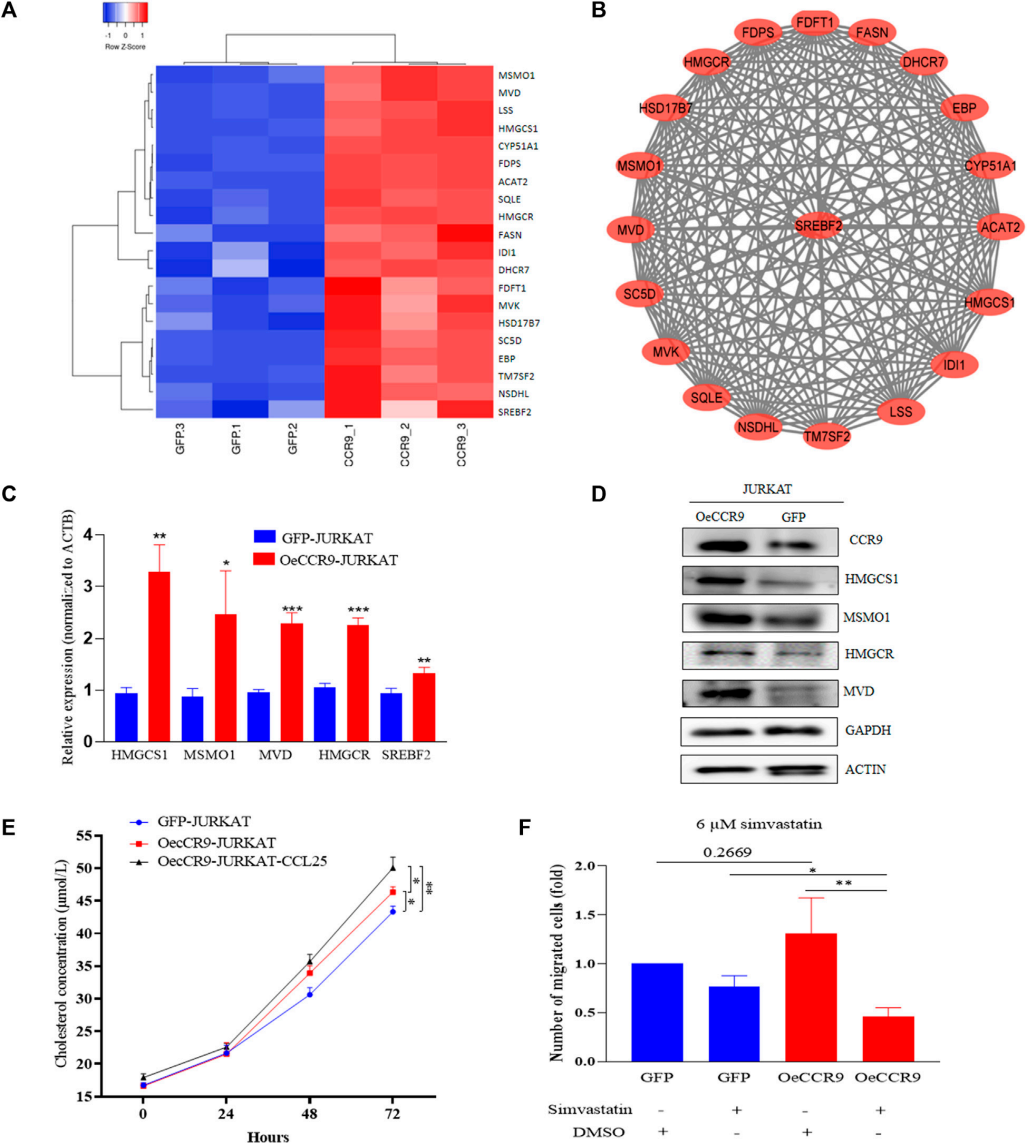
The overexpression of CCR9 is associated with increased cholesterol biosynthesis in T-ALL. **(A)** A heatmap of the DEGs associated with cholesterol biosynthesis pathways. **(B)** The protein-protein interaction network of the cholesterol biosynthesis pathway genes visualized with Cytoscape shows a strong interaction among these genes. **(C)** Quantification of the expression of MSMO1, HMGCS1, MVD, HMGCR and SREBF2 in OeCCR9-JURKAT cells vs. GFP-JURKAT cells on mRNA level **(D)** The protein expression of CCR9, HMGCS1, MSMO1, HMGCR and MVD was analyzed by Western blot. GAPDH and ACTIN were used as loading control. The corresponding antibodies for CCR9, HMGCS1, and MSMO1 were diluted in (1:1000). Antibodies for MVD and HMGCR were diluted at 1:200 and 1:500 ratios, respectively. GAPDH and ACTIN antibodies were diluted at 1:3000 dilutions. Blots were incubated with their corresponding primary antibodies overnight at 4 C. The secondary antibodies used were diluted at 1:3000 followed by incubation for 2 h at room temperature. **(E)** Total cholesterol concentration detected at different time points between OeCCR9-JURKAT cells, OeCCR9-JURKAT cells supplemented with CCL25, and GFP-JURKAT control cells. **(F)** Assessment of cell migration of OeCCR9-JURKAT cells vs. GFP-JURKAT cells post simvastatin treatment as compared to mock. ∗*p* < 0.05, ∗∗*p* < 0.01, ∗∗∗*p* < 0.0001 by 2-tailed Student’s *t*-test.

Next, we assessed the role of these DEGs in the cholesterol biosynthesis pathway. The genes belonging to the cholesterol biosynthesis pathway that are upregulated with CCR9 overexpression are represented in a heatmap (Figure 8A). A protein-protein interaction (PPI) network of the DEGs associated with the cholesterol biosynthesis pathway was retrieved from the online resource STRING and visualized with Cytoscape (Figure 8B). The network shows a closer association of these genes with each other. To corroborate the elevated expression of the cholesterol biosynthesis pathway genes in our genome-wide transcriptome data, we checked the expression of the rate limiting enzyme, HMGCR, and three main genes, including MSMO1, HMGCS1, and MVD at mRNA level, which showed a significant upregulation of these genes (Figure 8C). Notably, we also verified the elevated expression of the master regulator of the cholesterol biosynthesis pathway, SREBF2 (Madison, 2016) (Figure 8C). To assess the expression of these genes at protein level, Western blotting was performed. We confirmed the overexpression of these genes with CCR9 upregulation as compared to GFP control (Figure 8D). Furthermore, we screened the expression of these genes in GSE48558 (Cramer-Morales et al., 2013) and found a relatively higher expression of MSMO1, HMGCS1, MVD,SREBF2, and HMGCR in T-ALL cell lines and patients (Supplementary Figure S7A). Moreover, the analysis of GSE26713 (Homminga et al., 2011) also showed an increased expression of MSMO1,HMGCS1, SREBF2, and HMGCR in T-ALL cell lines and patients (Supplementary Figures S7A, B), but MVD showed no significant difference (Supplementary Figure S6B). In addition, the increased expression of MSMO1, HMGCS1, MVD, and SREBF2 across solid tumors vs. normal tissues based on the analysis of TCGA datasets (Supplementary Figures S8A–D) further signifies the crucial role of these metabolic genes in multiple cancers.

To check whether CCR9-induced upregulation of the cholesterol biosynthesis genes has an impact on the total cellular cholesterol level, we detected the total cholesterol level in the OeCCR9-JURKAT and GFP-JURKAT cells. Notably, we found a slight increase in the cholesterol concentration of the OeCCR9-JURKAT cells (46.35 μmol/L) as compared to the GFP-JURKAT cells (43.46 μmol/L) (Figure 8E). Notably, TC levels were increased further (50.71 μmol/L) when CCL25 was added to stimulate CCR9-overexpressing JURKAT cells. These findings support the notion that the upregulation of CCR9 could enhance total cellular cholesterol production in T-ALL cells, and this effect is further potentiated with the addition of CCL25.

To investigate the effect of increased cholesterol biosynthesis on the aggressiveness of the JURKAT cells, we inhibited the total cholesterol biosynthesis by treating the cells with varying concentrations of simvastatin (3 μM, 6 μM, 12 μM, and 24 μM), a hypocholesterolemic drug that blocks the activity of HMGCR receptor (Corsini et al., 1995). We observed that a concentration above 10 μM compromised the viability of the cells leading to cell death (data not shown). Subsequently, we selected a 6 μM concentration of treatment for the chemotaxis assay and observed a notable decrease in the migration of OeCCR9-JURKAT cells with simvastatin as compared to the mock treatment (Figure 8F). Moreover, immunohistochemical analysis of the main transcription factor, SREBF2, in the mice tissues xenografted with OeCCR9-JURKAT cells and GFP-JURKAT cells revealed a relatively increased expression of SREBF2 in the liver tissue of the OeCCR9-JURKAT mice as compared to the GFP counterpart (Supplementary Figures S9A, B). However, no distinction in the expression of SREBF2 was observed in the spleen tissue (Supplementary Figures S9C, D) between the two groups, suggesting enhanced cholesterol production in the liver, the organ primarily responsible for cholesterol production. In addition to these experiments, we used the https://tnmplot.com/analysis/ database, which contains ChIP-seq and RNA-seq data of various tumors vs. normal tissue, to check the correlation between CCR9 and SREBF2. A significant positive correlation (*R* = 0.43, p value = 0.00) between CCR9 and SREBF2 in ALL was observed (Supplementary Figure S9D).

Taken together, these results suggest that CCR9 overexpression promotes cholesterol biosynthesis in JURKAT cells by upregulating the candidate genes, and blocking the cholesterol biosynthesis pathway with simvastatin reduces the aggressiveness of the CCR9-overexpressing JURKAT cells.

## 4 Discussion

T-ALL is a hematological malignancy characterized by the abnormal expansion of immature lymphoid cells (Vadillo et al., 2018). The molecular mechanism of the leukemic cells’ infiltration into distant organs is a complex process that remains largely unknown. The *in vitro* and *in vivo* application of T-ALL cells remains a standard toolkit in cancer biology to understand the molecular mechanism of T-ALL. But these cell lines differ in their tumorigenic potentials and often demonstrate dissimilar T-ALL-inducing potentials when utilized in murine models.

This study reports the distinct T-ALL initiation and progression patterns of MOLT4 and JURKAT cell lines. Our findings suggest that MOLT4 cells show a more aggressive T-ALL phenotype as compared to JURKAT cells in the NTG mice, characterized by increased tumor tissue infiltration as indicated by FC, HE staining, and IHC analyses. Unlike the liver and spleen, the difference in T-ALL cell infiltration into the brain was not remarkable between the groups. The central nervous system is one of the sanctuary sites of ALL, with 5%–8% of the patients possessing CNS pathology causing damage to the cranial nerves and infiltration into the meninges (Jabbour et al., 2005; Jabbour et al., 2015), suggesting that not all the ALL patients present with CNS involvement. Gene expression profiling revealed enrichment of DEGs in vital cancer-associated signaling pathways such as PI3K-Akt and MAPK-ERK, extracellular matrix reorganization, DNA replication and cell cycle regulation, PTEN regulation, and RHO GTPases formins. These signaling pathways have been previously reported in the pathogenesis of leukemia (Steelman et al., 2011). Particularly, PI3K-Akt is the most predominant activated signaling pathway in more than 70% of T-ALL patients (Silva et al., 2008). In T-ALL, activation of the PI3K/AKT pathway is commonly observed and contributes to the survival and proliferation of leukemic cells. Inhibition of the PI3K/AKT pathway has been shown to be an effective therapeutic strategy for T-ALL.

CCR9, an upregulated gene in the list of DEGs, belongs to the G protein-coupled receptor family and is of great clinical relevance in T-ALL, particularly because of its relevance to tumor infiltration and metastasis (Tu et al., 2016; Li et al., 2021). The CCL25/CCR9-mediated RhoA-ROCK-MLC/ezrin axis has been reported to promote T-ALL metastasis mainly in MOLT4 cells (Zhang et al., 2011). Remarkably, the aberrant expression of CCR9 impacted the invasion and migration of T-ALL cells in our functional genetic analysis *in vitro*. Further, the overexpression of CCR9 led to a drastic increase in tumorigenic activity in the NTG mice characterized by a surge in JURKAT cell infiltration to the spleen and liver, as well as marked cell expansion in BM and PB.

T-ALL, being a highly proliferative malignancy, requires the rewiring of cholesterol biosynthesis metabolic pathways to sustain the rapid growth and proliferation of the leukemic cells (Zhao et al., 2019). In addition, the role of cholesterol biosynthesis is widely appreciated for promoting the aggressiveness of multiple cancer types (Ding et al., 2019). Among the common mechanisms used by cancer cells to alter intracellular cholesterol are the upregulation of the cholesterol biosynthesis pathway genes and the repression of the cholesterol efflux protein (Luo et al., 2020). In this study, gene expression profiling of OeCCR9-JURKAT cells showed the upregulation of several genes belonging to the cholesterol biosynthesis family, including SREBF2 and the rate limiting enzyme HMGCR, and other genes including HMGCS1, HMGCR, MVK, and MVD. The potential significance of these cholesterol biogenesis-related genes in various malignancies has been illustrated by (Ershov et al., 2021). The clinical relevance of these genes was further verified in T-ALL, showing enhanced expression in T-ALL cell lines and T-ALL patients. However, the precise oncogenic function of these genes in T-ALL remains mysterious. In a recent study, characterization of the metabolome and transcriptome in ETP-ALLs, a discrete group of T-cell leukemia associated with relapse and poor prognosis, revealed an elevated biosynthesis of phospholipids and sphingolipids in ETP-ALL as compared to T-ALL (Rashkovan et al., 2022). Moreover, Following HMGCR inhibition with pitavastatin treatment, an attenuated oncogenic AKT1 signaling was evident along with the suppression of MYC signaling pathway expression via the loss of chromatin accessibility at the leukemia stem cell-specific long-range MYC enhancer. HMGCR inhibition blocked cell proliferation, dampened cell viability, and compromised cell growth *in vitro* (Rashkovan et al., 2022). This is consistent with our results showing reduced migration of the OeCCR9-JURKAT cells following simvastatin treatment.

The existing data shown in this study lacks *in vivo* confirmation of the antileukemic effect of statin treatment. Several lines of investigation confirm the notion that inhibiting the rate-limiting enzymes of the cholesterol production pathway with statin exhibits profound antileukemic functions (Rashkovan et al., 2022). For instance, statin treatment induced selective autophagy in the leukemic cell lines by attenuating the phosphorylation of Akt levels in the lipid rafts along with a reduction in the activation of the vital autophagy suppressor mTOR pathway and its associated substrate, ribosomal p70S6 (Vilimanovich et al., 2015). Apart from these preclinical studies, several clinical trials have suggested the anti-leukemic activities of statins (Minden et al., 2001; Li et al., 2003; van der Weide et al., 2009). In addition, statin administration has been documented to improve the efficacy of standard therapy in acute myeloid leukemia (Kornblau et al., 2006; Advani et al., 2014). Sheen et al. investigated the *in vitro* effects of six different statins on ALL cells and showed that simvastatin resulted in ALL cell death by inducing apoptosis and exerted a synergistic effect in combination with other cytotoxic drugs, including vincristine, doxorubicin, and dexamethasone (Sheen et al., 2011). The similar antitumor function of statin on ALL cells, characterized by cell-cycle arrest, and induction of apoptosis mainly by upregulating BAX, p21, and p27 cells and downregulating cyclin D1, BCL-2, and p-Akt expression, has also been documented in another study (Wang et al., 2018). Dysregulated cholesterol biosynthesis has been associated with T-ALL drug resistance. Subsequently, inhibition of this pathway with simvastatin displayed a strong combined cytotoxic effect with gluocorticoids in resistant cells (Samuels et al., 2014b). In a preclinical setting, a T-ALL *in vivo* model developed from patient-derived T-ALL xenografts showed aberrant lipid and cholesterol metabolism as potential drivers of drug resistance and suggested simvastatin as a potential treatment regimen to overcome drug resistance (Samuels et al., 2014a).

The cholesterol biosynthesis pathway is under the strict regulation of SREBP2 and liver X receptors (LXRs). In other instances, metabolic reprograming involves the synergistic interaction of SREBF2 with other transcription factors such as early growth response element 1 (EGR1) (Gokey et al., 2011) and the nuclear receptor RAR-related receptor gamma (RORγ) to promote cholesterol biosynthesis, particularly in cancer (Cai et al., 2019). The precise mechanism by which chemokines induces cholesterol biosynthesis pathway remains largely unknown. Chemokines can either directly act on SREBP2 to activate the pathway, as evident in the tropism of breast cancer to the lungs, where CCL2/CCL7 produced from the lung fibroblast stimulates the synthesis of cholesterol by activating SREBF2 in lung-colonizing breast cancer cells to fuel the metastatic niche (Han et al., 2022). Consistently, we observe an increase in SREBF2 expression with CCR9-upregulation not only on the transcript level *in vitro* but also on the protein level in NTG mice liver tissue, suggesting the potential activation of SREBF2 to facilitate *de novo* cholesterol biosynthesis. Gaining mechanistic insights into the molecular mechanism of CCR9-SREBF2 in boosting cholesterol biosynthesis would be an exciting future research avenue in the relatively new era of T-ALL cholesterol metabolism.

## 5 Conclusion

In summary, we found that MOLT4 cells possess relatively higher aggressive tumorigenic potentials, characterized by their robust organ infiltration, as compared to JURKAT cells. Transcriptome profiling revealed several DEGs with enrichment in vital oncogenic pathways. Particularly, CCR9 not only facilitated the migration and invasion of cells *in vitro* but also promoted organ infiltration in the mouse model. CCR9 overexpression was associated with increased cellular cholesterol production, mainly due to the increased expression of the core regulatory genes of the cholesterol biosynthesis pathway. The enhanced cholesterol biosynthesis rate in turn promoted the aggressiveness of the JURKAT cells, which was markedly repressed with simvastatin administration. The findings in the current study reveal a novel mechanism for cholesterol biosynthesis that supports T-ALL cell migration and invasion. These findings propose that inhibiting the CCR9-induced cholesterol biosynthesis pathway with statin may pave the way for the development of effective therapeutic strategies to treat T-ALL progression.

## Summary

T-ALL is a form of hematological cancer derived from the early T-cell progenitor. The disease arises as a result of the accumulation of genomic lesions affecting signaling pathways involved in cell growth, proliferation, survival, and differentiation of thymocytes. In order to understand the biological mechanism of T-ALL, *in vitro* T-ALL cell lines are commonly employed. However, a comprehensive comparison of two common T-ALL cell lines, MOLT4 and JURKAT cells, for T-ALL development is not yet available. We compared MOLT4 and JURKAT cells for their T-ALL inducing potentials and found that MOLT4 cells exhibited a relatively increased aggressive leukemic phenotype in mice as compared to JURKAT cells. We examined the molecular characteristics of two cell lines that could lead to differences in cancer development. Transcriptional profiling of MOLT4 and JURKAT cells revealed significant changes in the expression of several genes involved in important signaling pathways, including CCR9. Notably, the aberrant expression of CCR9 impacted the migration and invasion of the T-ALL cell lines *in vitro*. In addition, higher expression levels of CCR9 also promoted T-ALL progression *in vivo*. Transcriptome analysis revealed that the overexpression of CCR9 promoted cholesterol biosynthesis in JURKAT cells. The altered lipid biosynthesis facilitates fatty acid synthesis, which is crucial to maintaining lipid homeostasis for membrane production and lipid-based protein posttranslational modifications in the rapidly proliferating tumor cells.

## Data Availability

The transcriptome raw data generated in this study have been deposited to NCBI SRA database (BioProject ID PRJNA897746) and can be accessed through https://www.ncbi.nlm.nih.gov/bioproject/?term=PRJNA897746.

## Supplementary material

SUPPLEMENTARY FIGURE S1 Analysis of the organ weight and tissue infiltration in mice in each group **(A)** Comparison of the size of the spleen organ obtained from mice in three groups. The spleen weight of the mice in each group was also calculated. **(B)** Representation of the size and weight of the liver in each group. **(C)** H.E. staining of the tissue sections derived from the liver and spleen of mice injected with PBS, MOLT4, and JURKAT, respectively.

SUPPLEMENTARY FIGURE S2 Pathway analysis of the DEGs between MOLT4 and JURKAT **(A)** DisGeNET gene ontology (GO) analysis of the DEGs **(B)** The GO shows the enrichment of the DEGs in various pathways. **(C)** The enrichment of DEGs in a cellular component, molecular function, and biological process categories in GO function.

SUPPLEMENTARY FIGURE S3 Distribution of the DEGs across the GSE datasets **(A)** Volcano plot representing the DEGs in the GSE33315 dataset **(B)** Volcano plot representing the DEGs in the GSE48558 dataset **(C)** Volcano plot representing the DEGs in GSE13159. The red and blue dots refer to the upregulated and downregulated genes, respectively. The log2FC value > 1 of CCR9 is mentioned in each dataset.

SUPPLEMENTARY FIGURE S4 Pictorial representation of the migration and invasion assays **(A)** Schematic illustration of the different parts of the transwell system used to assess the migration of the cells. Cells are seeded on the upper side of the transwell membrane in 1% FBS. To allow the migration of the cells, 10% FBS containing media or chemoattractant is added. **(B)** To allow the distinction between migration and invasion, an extracellular matrix component like matrigel coated matrix is added, which mimics a more complex physiological environment, to allow the invasion of the cells.

SUPPLEMENTARY FIGURE S5 Organ weight and HE staining analysis **(A)** a representative picture of the spleen size and weight between OeCCR9 and GFP-JURKAT cells. **(B)** A representative picture of the liver size and weight between OeCCR9 and GFP-JURKAT cells. **(C)** HE staining of the section obtained from the bone marrow, peripheral blood, liver, brain, and spleen of the mice in the two groups.

SUPPLEMENTARY FIGURE S6 Pathway analysis of the DEGs between OeCCR9- and GFP-JURKAT cells **(A)** The DisGeNET functional enrichment analysis of the DEGs shows the involvement of the DEGs in various diseases. **(B)** The KEGG functional enrichment analysis of the DEGs in metabolic pathways.

SUPPLEMENTARY FIGURE S7 Expression analysis of the MSMO1, HMGCS1, MVD, and SREBF2 across GSE datasets **(A)** Relative expression of MSMO1, HMGCS1, MVD, SREBF2, and HMGCR in T-cells vs. T-ALL cell lines and T-ALL patients across the GSE48558 dataset as compared to healthy bone marrow in the GSE13159 dataset samples, respectively. **(B)** Analysis of the GSE26713 for the relative expression of MSMO1, HMGCS1, MVD, SREBF2, and HMGCR in normal bone marrow samples vs. T-ALL. **p* < 0.05, ***p* < 0.01, ****p* < 0.0001 by 2-tailed Student’s t-test.

SUPPLEMENTARY FIGURE S8 Expression analysis of the MSMO1, HMGCS1, MVD, and SREBF2 across the TCGA database **(A-D)** relative expression of MSMO1, HMGCS1, MVD, and SREBF2 in various solid tumors as compared to the corresponding normal tissue. The expression analysis is done using TCGA datasets.

SUPPLEMENTARY FIGURE S9 IHC of SREFBF2 in the liver and spleen tissue of mice transplanted with OeCCR9-JURKAT cells vs. GFP-JURKAT. IHC analysis of SREBF2 expression in liver and spleen tissue sections. **(B)** Representative images of the SREBF2 expression in liver of OeCCR9-JURKAT-NTG and GFP-JURKAT-NTG mice. **(B)** H-scores of the SREBF2+ cells in the liver of OeCCR9-JURKAT-NTG and GFP-JURKAT-NTG mice. **(C)** Representative images of the SREBF2 expression in spleen of OeCCR9-JURKAT-NTG and GFP-JURKAT-NTG mice. **(D)** H-scores of the SREBF2+ cells in the liver of OeCCR9-JURKAT-NTG and GFP-JURKAT-NTG mice. **(E)** TNMplot database was used to screen the correlation between CCR9 and SREBF2 in T-ALL. We selected CCR9: gene **(A)** vs. SREBF2: gene **(B)**, T-ALL: tissue, Tumor: sample type, and Spearman: correlation method analysis.

SUPPLEMENTARY TABLE S1 List of upregulated genes.

SUPPLEMENTARY TABLE S2 List of downregulated genes.

SUPPLEMENTARY TABLE S3 KEGG pathway enrichment analysis of the DEGs.

SUPPLEMENTARY TABLE S4 GO enrichment analysis of the DEGs.

SUPPLEMENTARY TABLE S5 list of selective DEGs following log2FC < 7, *p*-value = smallest.

SUPPLEMENTARY TABLE S6 GO enrichment analysis of the DEGs between the GFP-JURKAT and OeCCR9-JURKAT cells.

## Abbreviations

BM, bone marrow; CCR9, CC chemokine receptor 9; CCL25, CC chemokine ligand 25l; DEGs, Differentially expressed genes; DO, Disease ontology; GEO, Gene Expression Omnibus; GO, Gene ontology; HE, hematoxylin and eosin; HMGCS1, 3-hydroxy-3-methylglutaryl-CoA synthase 1; HMGCR, 3-hydroxy-3-methylglutaryl-CoA reductase; IHC, Immunohistochemistry; KEGG, Kyoto encyclopedia of gene and genome; MVD, Mevalonate Pyrophosphate decarboxylase; MSMO1, Methyl sterol monooxygenase 1; PB, peripheral blood; PBS, Phosphate-buffered sa-line; qRT-PCR, Quantitative real-time PCR; SREBF2, Sterol regulatory element-binding factor 2; STRING, Search Tool for the Retrieval of Interacting Genes/Proteins; shRNA, short hairpin RNA; siRNA, Small interfering RNA; NTG, NOD-PrkdcscidIL2rgdull; T-ALL, T-cell acute lymphoblastic leukemia.
