# Supplementary material for: CCR9 overexpression promotes T-ALL progression by enhancing cholesterol biosynthesis
Source: Front Pharmacol. 2023 Sep 6;14:1257289. doi: 10.3389/fphar.2023.1257289 (PMC10512069; doi:10.3389/fphar.2023.1257289)
Supplement: Supplementary file 2 [file Presentation9.pptx]

## Slide 1
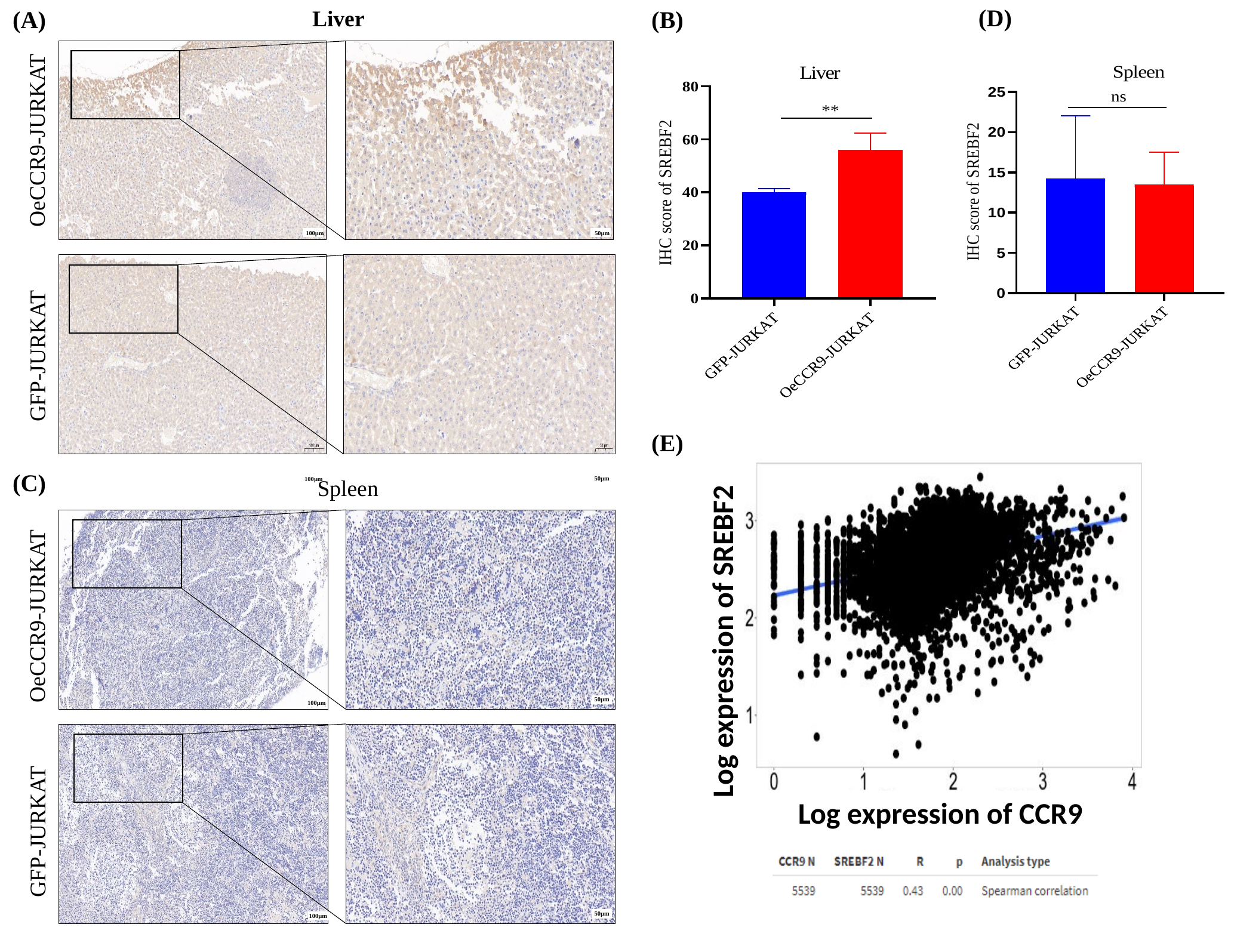

(A)
Liver
(B)
(D)
OeCCR9-JURKAT
100µm
50µm
GFP-JURKAT
(E)
Log expression of SREBF2
Log expression of CCR9
(C)
Spleen
50µm
100µm
OeCCR9-JURKAT
50µm
100µm
GFP-JURKAT
50µm
100µm
