# Supplementary material for: CCR9 overexpression promotes T-ALL progression by enhancing cholesterol biosynthesis
Source: Front Pharmacol. 2023 Sep 6;14:1257289. doi: 10.3389/fphar.2023.1257289 (PMC10512069; doi:10.3389/fphar.2023.1257289)
Supplement: Supplementary file 4 [file Presentation1.ppt]

## Slide 1
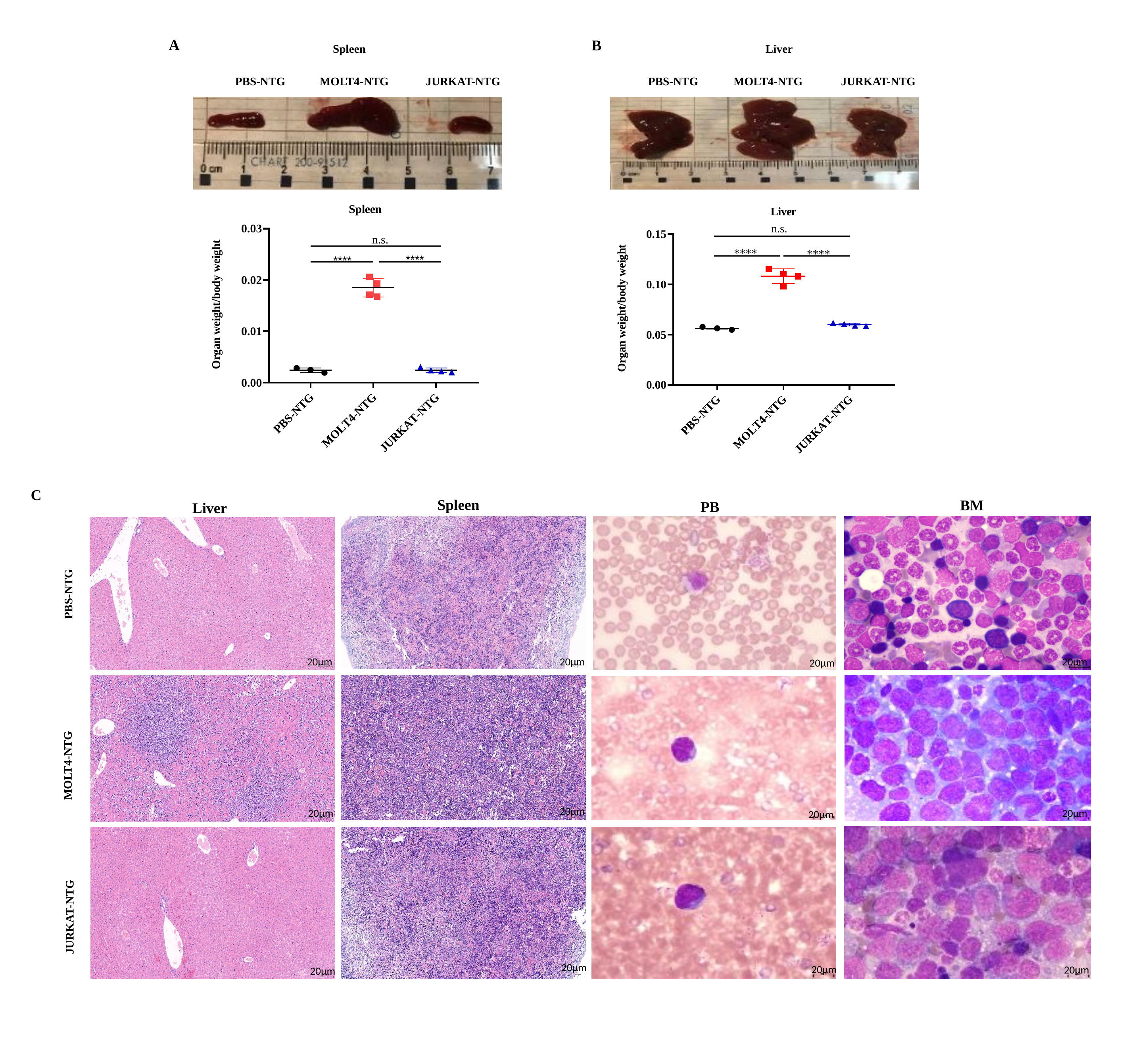

A
B
Spleen
Liver
MOLT4-NTG
JURKAT-NTG
PBS-NTG
PBS-NTG
MOLT4-NTG
JURKAT-NTG
C
Spleen
BM
PB
Liver
PBS-NTG
PBS
20µm
20µm
20µm
20µm
MOLT4-NTG
20µm
20µm
20µm
20µm
JURKAT-NTG
20µm
20µm
20µm
20µm
