# Supplementary figures and images for: CCR9 overexpression promotes T-ALL progression by enhancing cholesterol biosynthesis
Source: Front Pharmacol. 2023 Sep 6;14:1257289. doi: 10.3389/fphar.2023.1257289 (PMC10512069; doi:10.3389/fphar.2023.1257289)

## Slide 1
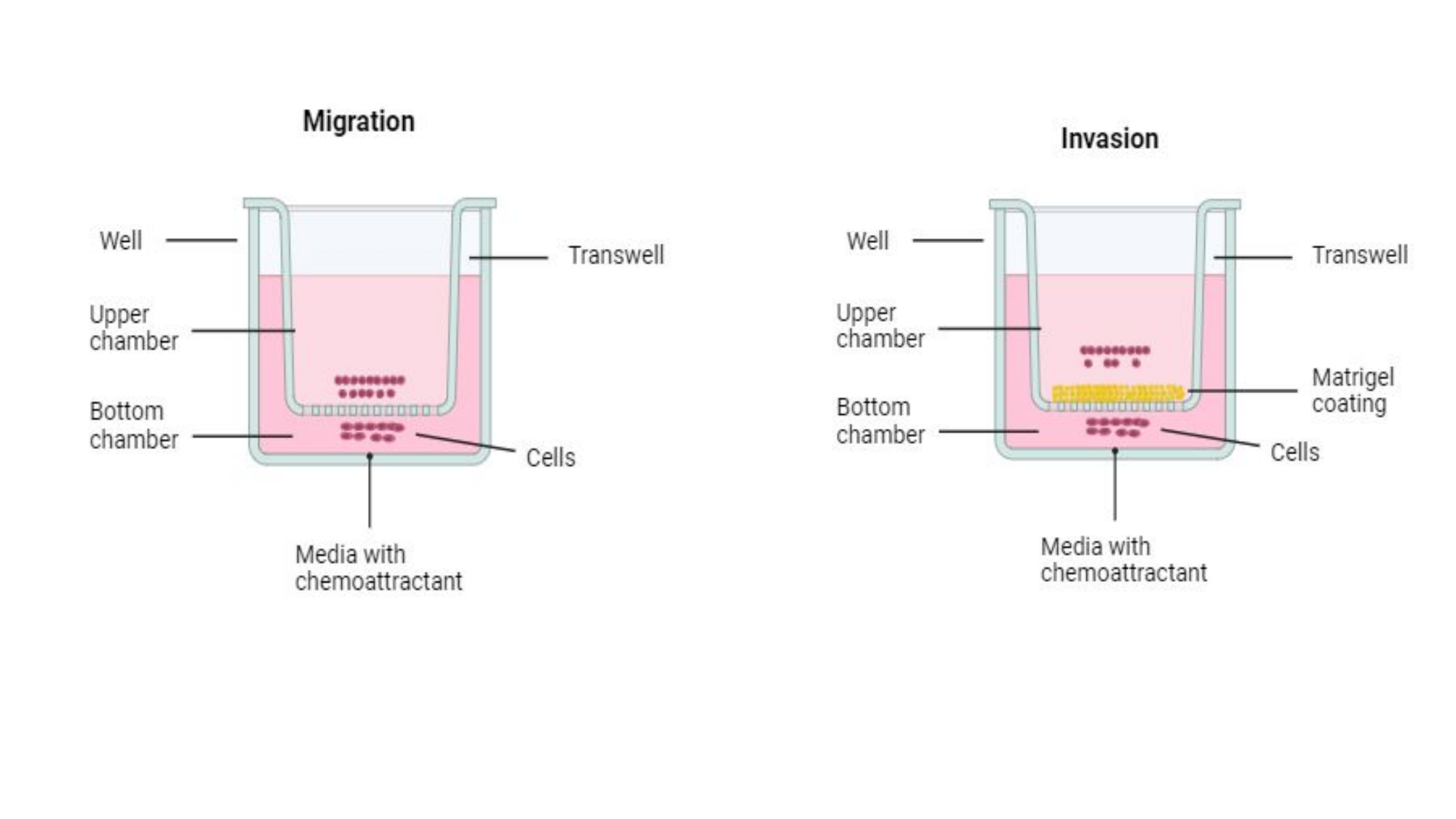

Supplement: Supplementary file 8 [file Presentation4.pptx]

## Slide 1
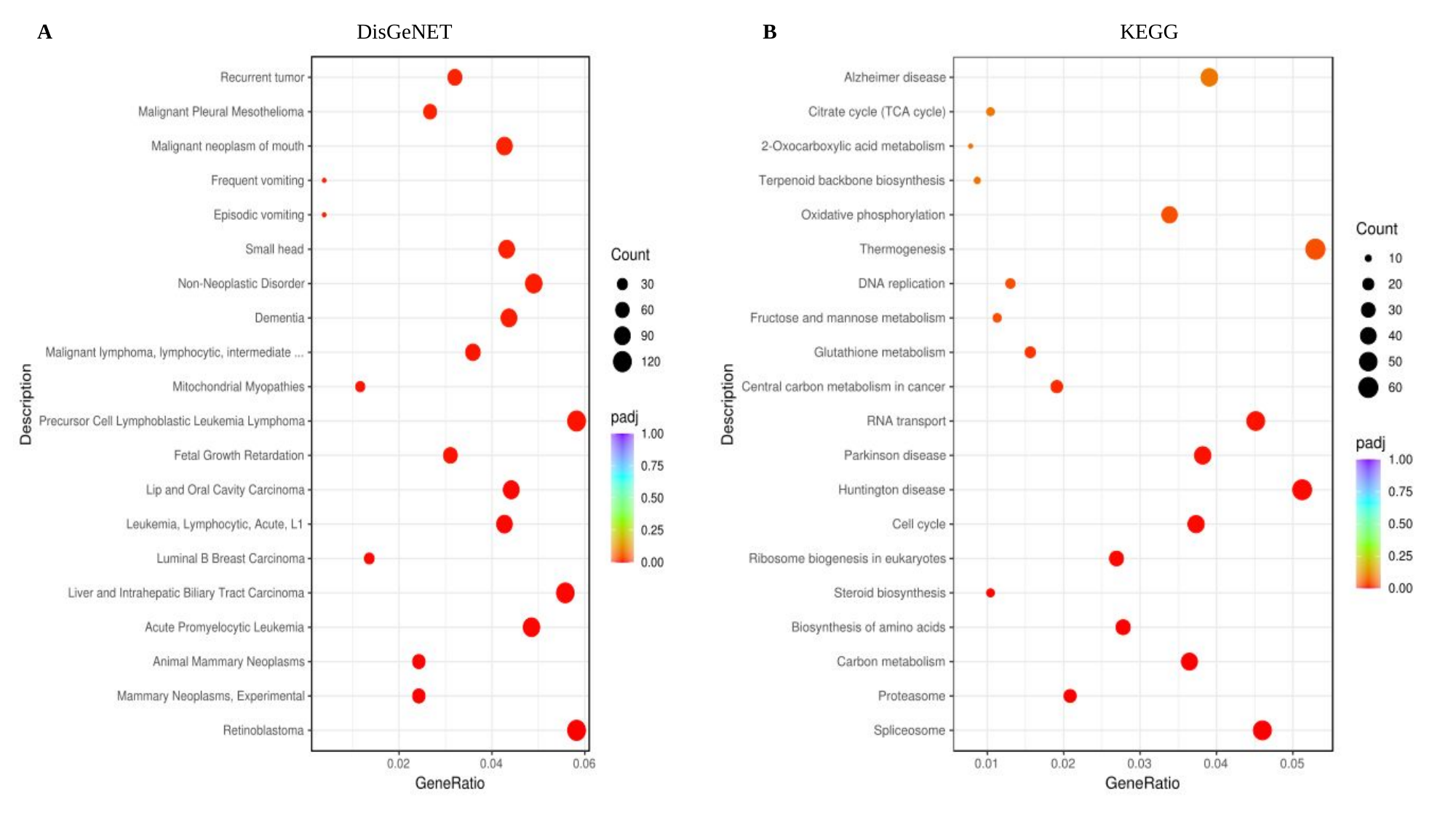

A
DisGeNET
B
KEGG

Supplement: Supplementary file 10 [file Presentation6.pptx]

## Slide 1
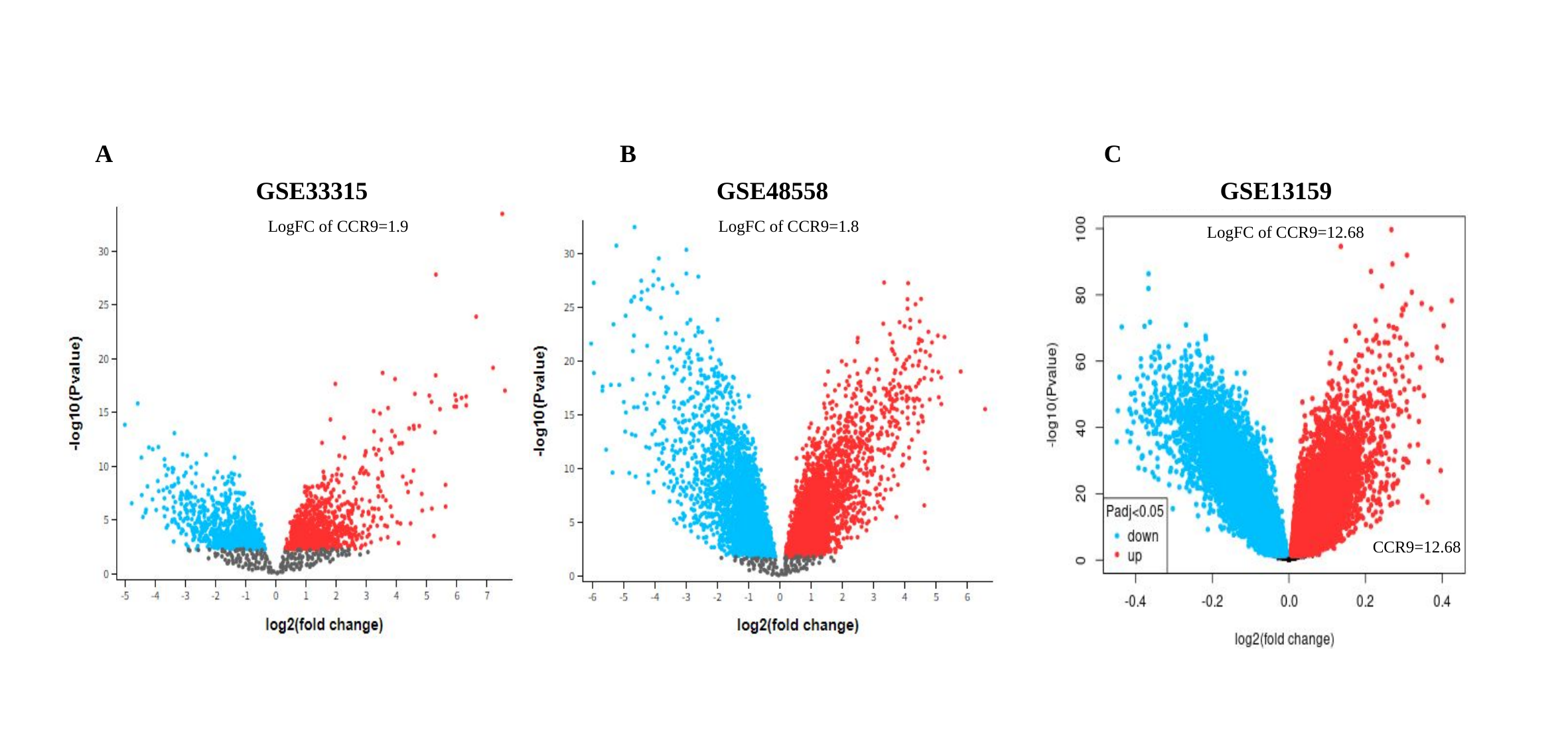

A
B
C
GSE33315
GSE48558
GSE13159
LogFC of CCR9=1.9
LogFC of CCR9=1.8
LogFC of CCR9=12.68
CCR9=12.68

Supplement: Supplementary file 13 [file Presentation3.pptx]

## Slide 1
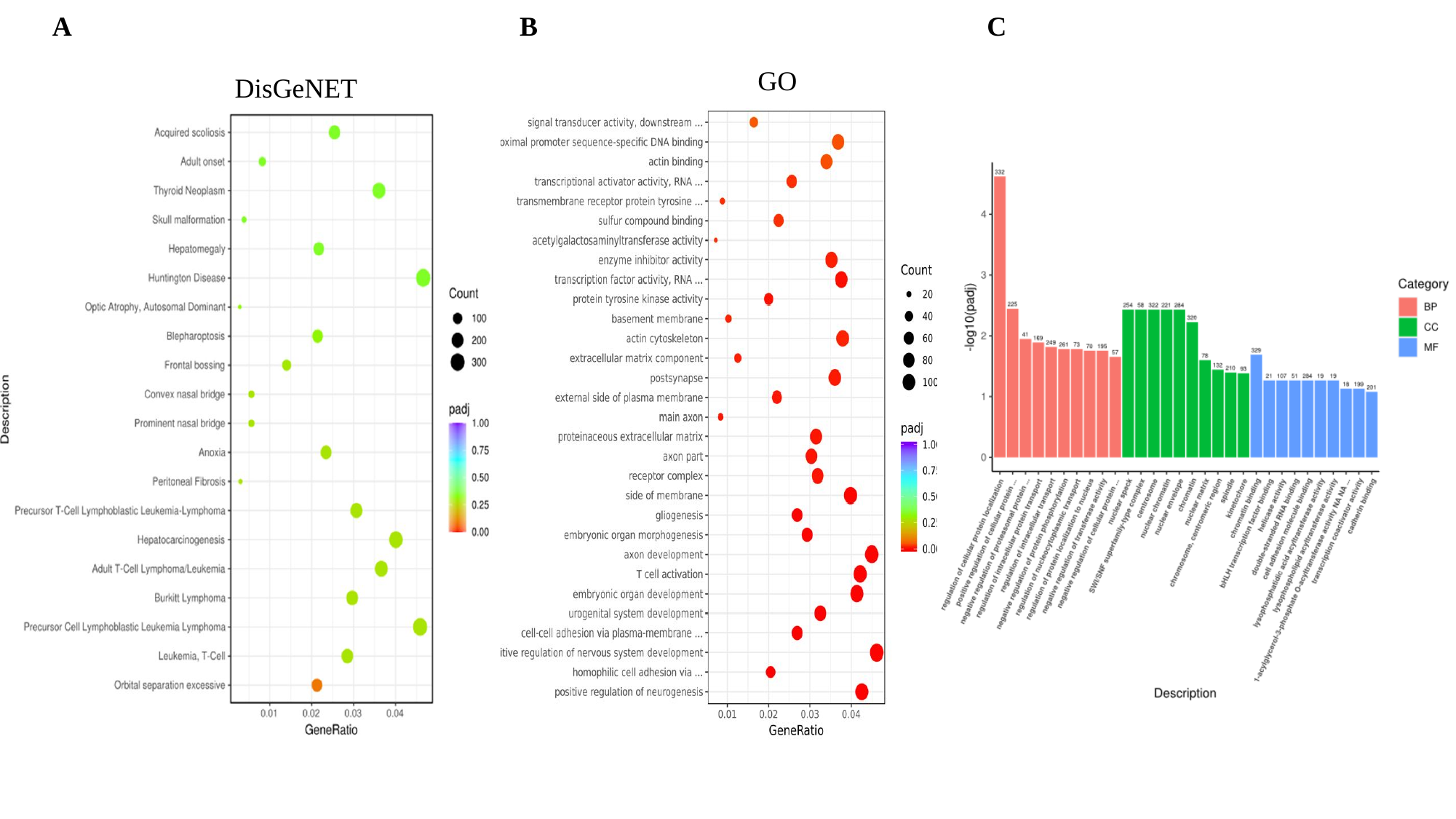

A
B
C
GO
DisGeNET

Supplement: Supplementary file 14 [file Presentation2.pptx]

## Slide 1
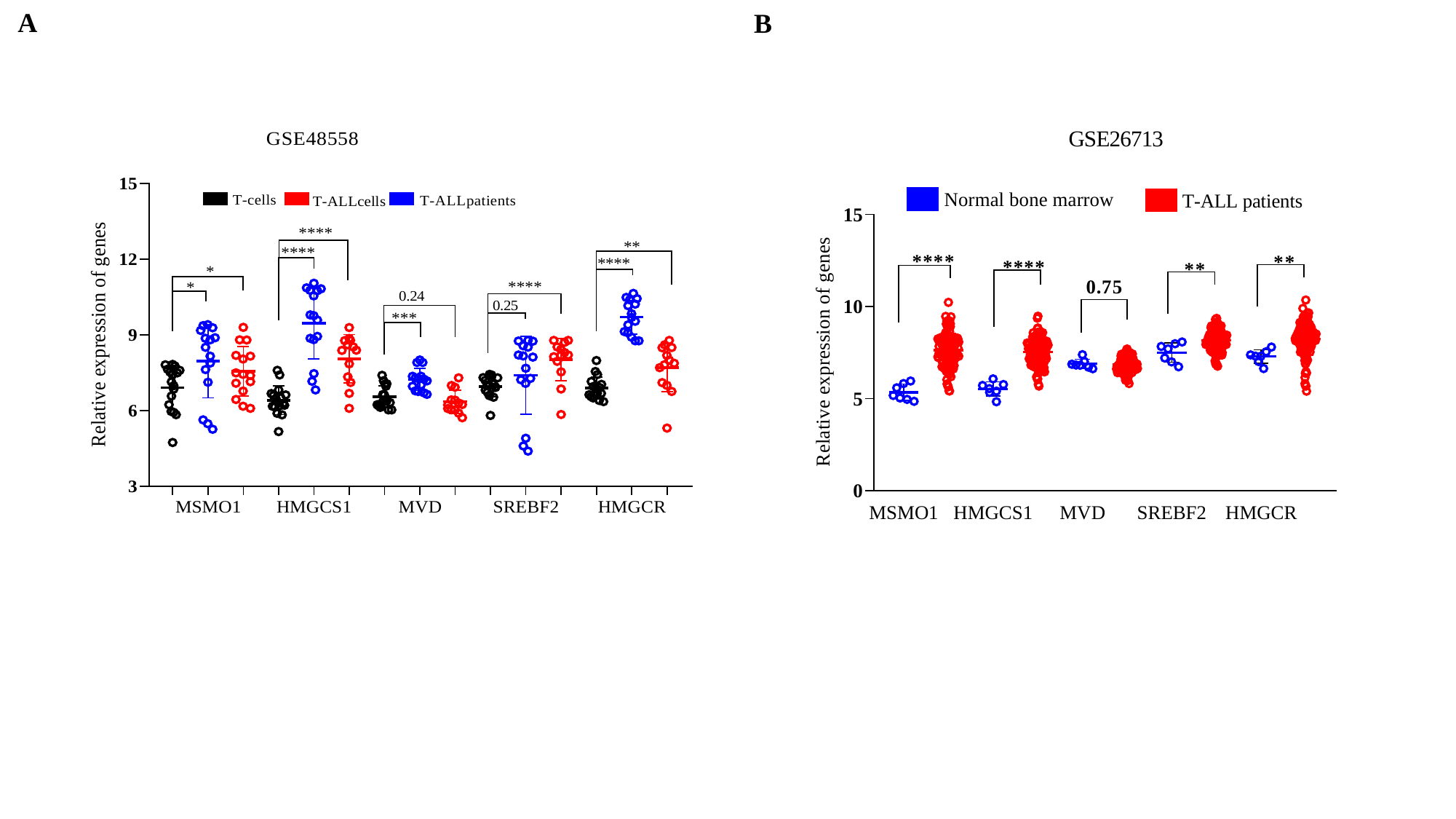

A
B

Supplement: Supplementary file 15 [file Presentation7.pptx]
