# Supplementary material for: CCR9 overexpression promotes T-ALL progression by enhancing cholesterol biosynthesis
Source: Front Pharmacol. 2023 Sep 6;14:1257289. doi: 10.3389/fphar.2023.1257289 (PMC10512069; doi:10.3389/fphar.2023.1257289)
Supplement: Supplementary file 11 [file Presentation5.ppt]

## Slide 1
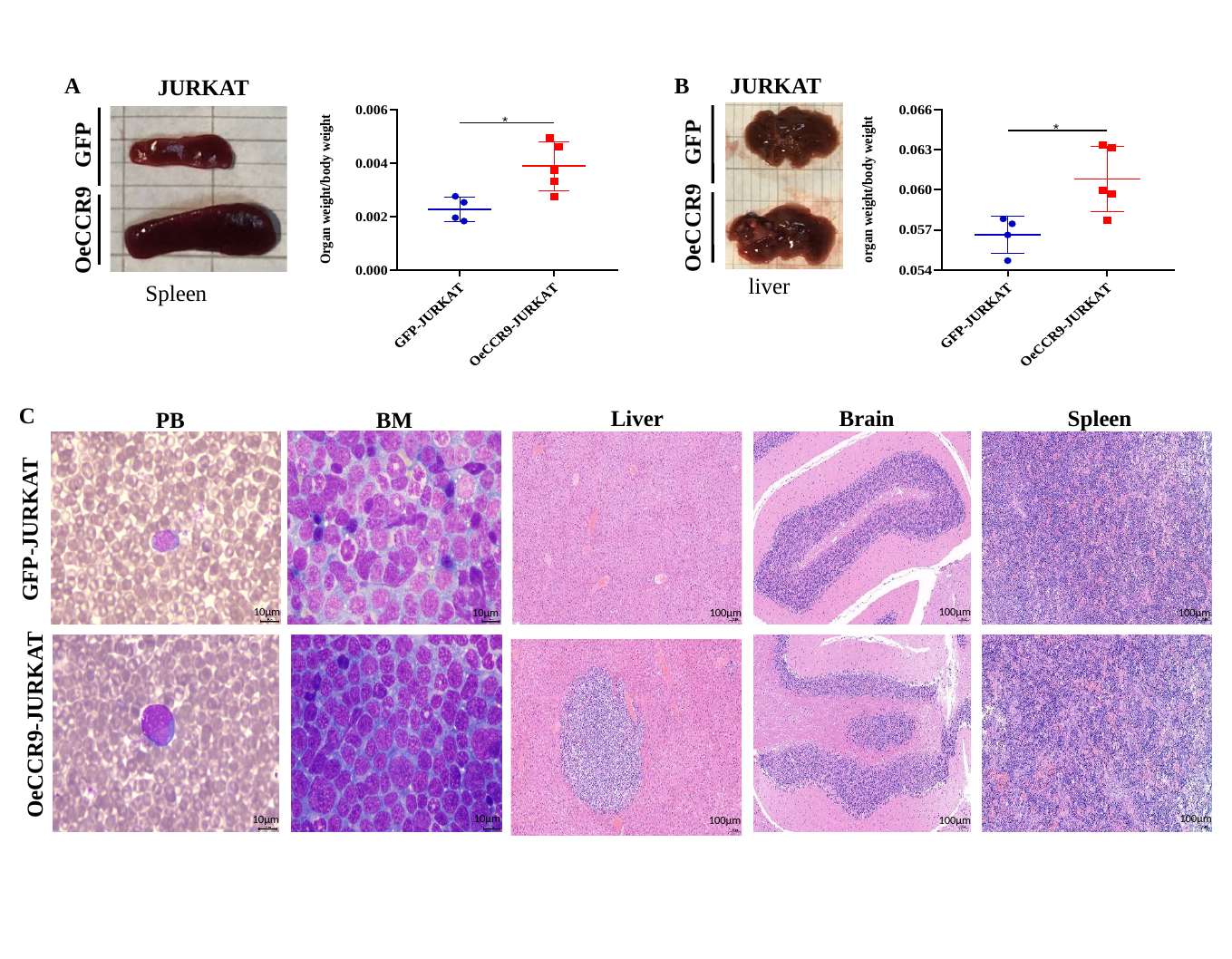

GFP
OeCCR9
JURKAT
GFP
JURKAT
OeCCR9
A
B
liver
Spleen
C
Spleen
Liver
Brain
PB
BM
GFP-JURKAT
10µm
100µm
10µm
100µm
100µm
OeCCR9-JURKAT
100µm
10µm
10µm
100µm
100µm
