# Supplementary material for: CCR9 overexpression promotes T-ALL progression by enhancing cholesterol biosynthesis
Source: Front Pharmacol. 2023 Sep 6;14:1257289. doi: 10.3389/fphar.2023.1257289 (PMC10512069; doi:10.3389/fphar.2023.1257289)
Supplement: Supplementary file 12 [file Presentation8.pptx]

## Slide 1
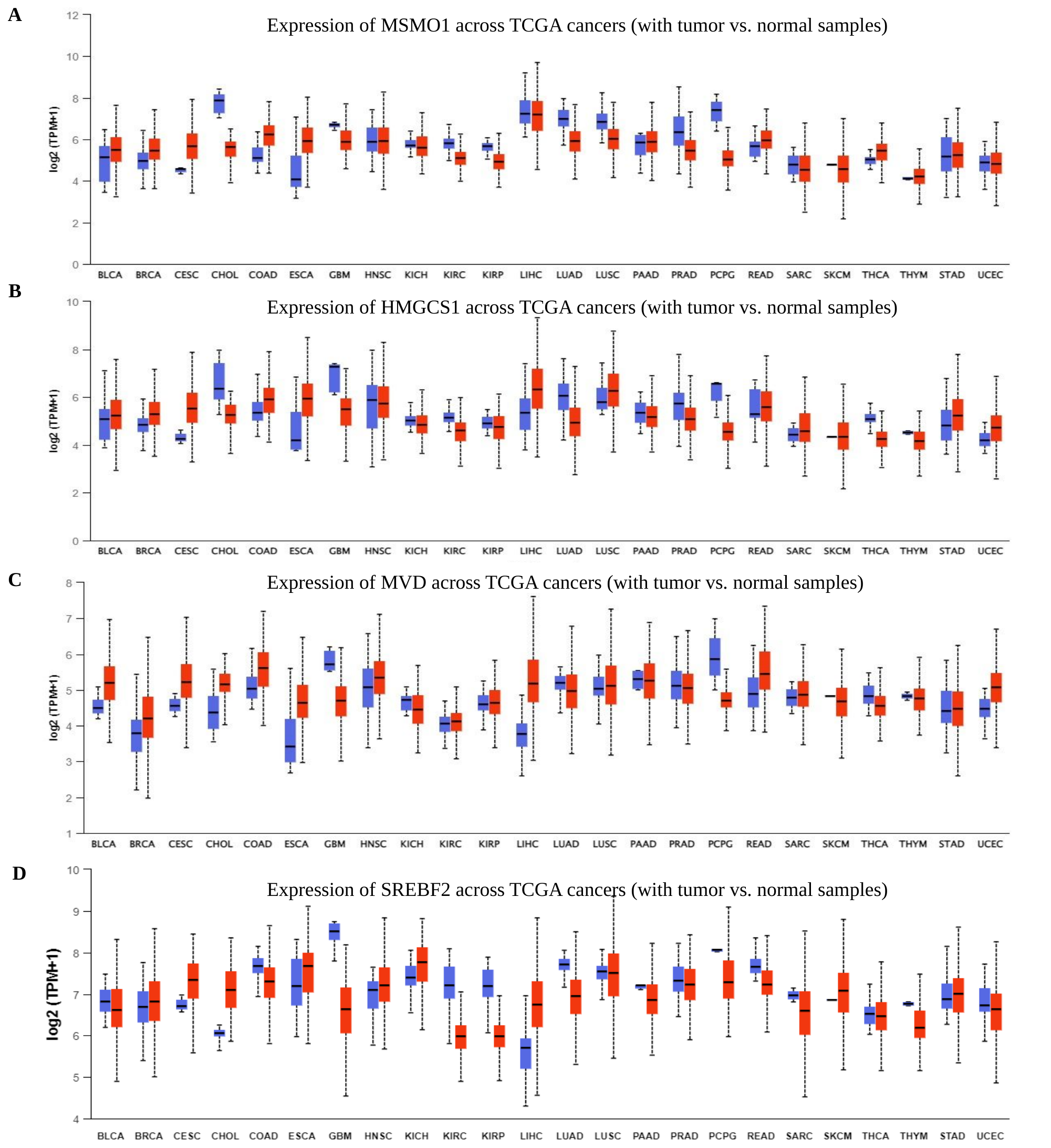

A
Expression of MSMO1 across TCGA cancers (with tumor vs. normal samples)
B
Expression of HMGCS1 across TCGA cancers (with tumor vs. normal samples)
C
Expression of MVD across TCGA cancers (with tumor vs. normal samples)
D
Expression of SREBF2 across TCGA cancers (with tumor vs. normal samples)
